# In Vitro Effects of the Three Actives IL-12 (5 CH), IFN-γ (6 CH), and TNF-α (5 CH) from the Micro-Immunotherapy Medicine 2LEID-N^®^ on Several Populations of Immune Cells

**DOI:** 10.3390/cimb48060566

**Published:** 2026-05-28

**Authors:** Camille Jacques, Flora Marchand, Mathias Chatelais, Elías Hurtado-Gaitán, Joana M. Buades, Ilaria Floris

**Affiliations:** 1 Preclinical Research Department, Labo’life France, Pescalis-Les Magnys, 79320 Moncoutant-sur-Sevre, France; 2 ProfileHIT, 3 Les Glands, 44680 Sainte-Pazanne, France; flora.marchand@profile-hit.com (F.M.); mathias.chatelais@profile-hit.com (M.C.); 3 AINIA, Centro Tecnológico, Parque Tecnológico de València, Av Benjamin Franklin, 5-11, 46980 Paterna, València, Spain; ehurtado@ainia.es; 4 Preclinical Research Department, Labo’life España, Av. des Raiguer, 7, 07330 Consell, Illes Balears, Spain; jm.buades@labolife.com

**Keywords:** micro-immunotherapy, immune modulation, respiratory infections, interleukin-12, tumor necrosis factor-α, interferon-γ

## Abstract

The micro-immunotherapy medicine (MIM) 2LEID-N^®^ was developed to sustain immune response, notably in the framework of respiratory infections. This pilot study investigated the potential effects of one capsule of this MIM, referred to as 2LEID-N-9 throughout the manuscript, containing IL-12 (5 CH), IFN-γ (6 CH), and TNF-α (5 CH). Phagocytosis and surface marker expression were assessed using flow cytometry, and cytokine secretion was assessed by ELISA. Cellular models included human monocyte-derived macrophages, peripheral blood mononuclear cells (PBMCs) from healthy donors, and THP-1 cells. Liquid chromatography coupled with high-resolution mass spectrometry (LC-HRMS/MS) was used to detect the actives. Compared with vehicle control, 2LEID-N-9 showed a trend towards the enhanced phagocytic activity of macrophages. In PBMCs, 2LEID-N-9 upregulated the secretion of several cytokines, including IL-2, IL-4, IL-13, IFN-γ, and TNF-α in both basal and CD3/CD28-stimulated conditions. Notably, a tendency towards increased secretion of TNF-α was found in LPS-stimulated THP-1 cells. The presence of the three actives, as assessed by LC-HRMS/MS, combined with the functional data, provide promising exploratory evidence of immunomodulatory effects and tendencies towards the stimulation of innate and adaptive immune cells, warranting further investigation.

## 1. Introduction

Ear, nose, and throat (ENT) infections comprise a diverse range of conditions that can affect either the upper (rhinitis, sinusitis, otitis, pharyngitis, epiglottitis, tracheitis) or lower parts (bronchitis, pneumonia) of the respiratory tract [1]. These infections are mainly due to microorganisms or pathogens, such as bacteria like *Streptococcus pyogenes* or various viruses (influenza A, coronaviruses, rhinoviruses, adenoviruses, and enteroviruses). Several factors are known to increase vulnerability to infection, to name a few: a low body mass index, susceptible populations, and interactions with pets [2]. Symptoms typically include a runny or blocked nose, sneezing, a sore throat, coughing, hoarseness, facial pressure, and a mild fever. As transmission between individuals occurs through direct physical contact, indirect contact (through the so-called “fomites”, which are contaminated objects), droplets, or aerosols inhalation [3], prevention measures are crucial. The latter includes hand and respiratory hygiene (medical mask, tissues), as recommended in healthcare settings [4], surface sanitation, wound disinfection, and, if symptoms persist, self-isolation. Current treatments mainly focus on symptom relief with over-the-counter decongestants, antihistamines, cough suppressants, expectorants, as well as plant-derived substances [5].

As the airways are continuously exposed to various external challenges, it is essential for the pulmonary tract’s immunity to be reactive. This ensures effective pathogen elimination and a quick return to homeostasis, while also preventing excessive, prolonged, or unnecessary immune responses. Multiple cells collaborate in order to manage immune responses in the respiratory tract, including regulatory and effector T-cells, natural killer (NK) cells, airway and interstitial macrophages, dendritic cells, and the airway epithelium [6,7].

As cytokines coordinate the physiological functions of the immune system, they consequently balance immune responses in health and disease, self-regulating immune responses through feedback loops [8]. Thus, their use in therapy is promising. In particular, micro-immunotherapy (MI) medicines (MIMs) could be interesting tools in the context of ENT treatment. Indeed, MIMs mainly employ human recombinant (hr) cytokines (and other signaling molecules), at low doses (LD) or ultra-low doses (ULD), expressed in Centesimal Hahnemannian dilutions (CH), to orient biological responses toward upregulation, or downregulation, as previously described [9,10]. These medicines were shown to display immune modulatory effects in vitro and in vivo, and could have interesting applications in clinics, under different inflammatory-mediated conditions, such as viral infections [11] or allergic diseases [12].

2LEID-N^®^, also referred to as 2LEID-N in the manuscript, is a MIM developed to sustain immune response, particularly in the case of infections from different origins (viral, bacterial, and/or fungal), such as ENT infections. 2LEID-N is a sequential MIM composed of ten different capsules, each with a specific composition of active substances formulated at LD or ULD. The current in vitro study investigated the effects of one specific capsule, the ninth of the sequence, thus referred to as 2LEID-N-9 within the current manuscript. This capsule combines three active substances: hr-interleukin (IL)-12 (5 CH), hr-interferon (IFN)-γ (6 CH), and hr-tumor necrosis factor (TNF)-α (5 CH). The rationale behind selecting the capsule of interest lies in its potential to modulate immune responses due to its specific composition, as further described: for instance, TNF-α (5 CH) and IFN-γ (6 CH), in association with other actives at LD/ULD in MIM, were previously seen to induce immune-stimulating effects in vitro, increase the phagocytosis capabilities of human monocyte-derived macrophages, and increase the secretion of chemokines involved in leukocyte migration and activation, such CXCL12 [13]. Furthermore, the presence of IL-12 at 5 CH combined with the two mentioned above is interesting, as IL-12 is known for its physiological role in promoting helper CD4^+^ T-cell type 1 (Th1)-type immune responses, which is essential for combating intracellular pathogens and influencing cell-mediated immunity [14]. In addition, TNF-α plays a critical role in inflammatory processes, especially in respiratory diseases [15], and IFN-γ is a pivotal player in antiviral defenses and immune regulation in this context [16]. Of note, the use of these actives in other MIM such as 2LEID, has shown interesting immunological effects in a preclinical in vivo model of viral respiratory infection [17]. Thus, to pursue investigations on the general immunological effects of MIM, and on the specific association of the three actives IL-12 (5 CH), IFN-γ (6 CH) and TNF-α (5 CH), this study focuses on the effects of the capsule 2LEID-N-9 from the MIM 2LEID-N. The hypothesis is that, 2LEID-N-9, based on its composition, may exert immune-stimulant effects.

Initially, the effect of 2LEID-N-9 was assessed on cells belonging to the innate side of immunity, especially macrophages. Thus, an in vitro assay measuring the phagocytic activity of macrophages against the yeast *Candida albicans* was performed. The research then focused on evaluating the impact of 2LEID-N-9 on the proliferation and expression of the activation marker human leukocyte antigen (HLA)-DR in human primary monocytes/macrophages, in normal basal/unstimulated culture conditions. In order to extend the cellular panel of the analysis, the proliferation and expression of another marker of cell activation, CD69, were appraised in human primary granulocytes and NK cells, still under the same basal/unstimulated conditions. The results of these experiments, performed in cells from the innate side of immunity, were enriched with an analysis of cytokine secretion in human peripheral blood mononuclear cells (PBMCs) derived from healthy donors, to delve into the link between innate and adaptive responses. In this part of the study, PBMCs from six healthy donors were treated with 2LEID-N-9 in basal culture conditions or were simultaneously exposed to CD3/CD28 stimulation, to mimic the T-cell receptor engagement. The secretion of five cytokines was appraised: IL-2, IL-4, IL-13, IFN-γ, and TNF-α. To gather more data on how 2LEID-N-9 influenced the secretion of TNF-α, an experiment was conducted using the human monocytic cell line THP-1.

Transitioning from these functional assays, this study turns to advanced analytical techniques using liquid chromatography coupled with high-resolution mass spectrometry (LC-HRMS/MS). As the active substances in MIM are expressed in CH, and considering that this nomenclature does not provide a direct correspondence to standard analytical mass units, there is growing interest in characterizing these formulations using modern platforms to bridge the gap between historical manufacturing conventions and contemporary proteomic identification standards.

The present study aims to provide direct analytical evidence regarding the molecular presence of the three actives within the tested MI formulation. The primary challenge was to develop a refined protocol to extract and identify the three specific cytokines from 2LEID-N-9 within the complex sugar crystalline matrix. This paper exclusively focuses on the qualitative detection and specific confirmation of these proteins using proteotypic peptides. This initial step serves as a proof of concept, demonstrating the feasibility of analytical identification of signaling molecules within MIM, establishing a materialistic foundation for the biological effects reported in the study.

## 2. Materials and Methods

### 2.1. Tested Items and Experimental Controls

The investigational product of this study is a Labo’life medicine, 2LEID-N^®^, referred to as 2LEID-N throughout the manuscript. 2LEID-N is a homeopathic medicinal product consisting of a sequence of ten different capsules containing sugar pillules, also called globules, impregnated with their unique ethanolic preparations of LD or ULD of immune factors, intended to be taken orally, according to the order indicated in the blister, in a fasted state. Out of the ten capsules composing the medicine, only one of them, 2LEID-N-9, the 9th of the sequence, was assessed in vitro in this study. The composition of the capsule is as follows: hr-IL-12 (5 CH), hr-IFN-γ (6 CH), and hr-TNF-α (5 CH). The tested items (2LEID-N-9 and vehicle (Veh.) capsules) were manufactured by Labo’life España, Avenida des Raiguer, 7, 07330 Consell—Mallorca, Spain, as previously described [10,17], and have been provided for investigational purposes. Previous publications have already described how Veh. capsules are produced to provide a suitable control for preclinical research [10,17,18]. In the experiments performed in the context of the current study, the sugar globules contained in each one of the tested capsules of 2LEID-N or Veh. capsules were freshly diluted in 50 mL or in 100 mL of culture medium to reach the final sugar concentration of 22 mM or 11 mM, respectively, depending on the experiments.

### 2.2. Phagocytosis Capability Assessment Experiment in Monocytes-Derived Macrophages

Peripheral blood mononuclear cells (PBMCs) were isolated from buffy coats of healthy volunteers, obtained from the Etablissement Français du Sang (EFS), using a standard Ficoll–Hypaque gradient method. Monocytes were separated from PBMCs via adherence to plastic surfaces for 2 h in serum-free macrophage-SFM medium (M-SFM; Gibco, Life Technologies, Carlsbad, CA, USA), designed specifically for macrophage culture, maintained at 37 °C in a humidified environment with 5% CO_2_. These monocytes were seeded and differentiated into macrophages in the presence of granulocyte macrophage colony-stimulating factor (GM-CSF) and IFN-γ, both supplied by Peprotech, Inc., Cranbury, NJ, USA. For *C. albicans* labeling, the yeast was heat-killed at 75 °C for 1 h, followed by labeling with pHRodo Red, succinimidyl ester (pHRodo Red, SE; ThermoFisher Scientific, Waltham, MA, USA), in line with the manufacturer’s instructions. The pHRodo dye becomes fluorescent under acidic conditions, such as those found in phagolysosomes, indicating effective phagocytosis. *C. albicans* was incubated with the pHRodo dye in sodium bicarbonate (final concentration of 20 μg/mL) for 1 h at room temperature in the dark. Excess dye was removed using a phosphate-buffered saline (PBS) washing step. The labeled *C. albicans* was then diluted in PBS and stored at 4 °C until needed. To confirm correct labeling, a test was performed in an acidic solution (pH 4) versus a neutral solution (pH 7.4). After a week of monocyte differentiation into macrophages, Veh. or 2LEID-N-9 were added to the cells for 24 h, at sugar concentrations of 22 mM. The following day, the culture medium and treatments were replaced before adding pHRodo-labeled *C. albicans* to the cells, which were then kept on ice to facilitate proper sedimentation. After 30 min on ice, the plate was analyzed using an Operetta imaging system over a 6 h period at 37 °C and 5% CO_2_, with data collected using Harmony Imaging Software 2019 (Perkin Elmer France, Villebon-sur-Yvette, Every, France). Each well was imaged every 8 min, with phagocytosis quantification conducted using Columbus 2.5.0 image analysis software. The experiment was performed once with *n* = 6 replicates per condition.

### 2.3. Assessment of Proliferation and Cell Surface Activation Marker Expression in Peripheral Blood Mononuclear Cell Subpopulations Using Flow Cytometry Under Baseline Conditions

Healthy volunteers were recruited by the French Blood Bank Center (Etablissement Français du Sang, [EFS], Pays de Loire, Nantes, France, www.efs.sante.fr, accessed on 02 February 2024), which operates under the French Ministry of Health.

In brief, PBMCs from four healthy donors, identified as a 21-year-old woman, a 35-year-old woman, a 57-year-old man, and a 37-year-old man, referred to as #A, #B, #C, and #D respectively, were freshly isolated via Ficoll^®^ gradient separation. They were then cultured for 48 h in the presence of either Veh. or 2LEID-N-9 (at 11 mM of sugar), under standard conditions: Roswell Park Memorial Institute (RPMI) 1640 medium supplemented with 2% inactivated human serum, 1 mM non-essential amino acids, 1 mM pyruvate, and 10 mM 4-(2-hydroxyethyl)-1-piperazineethanesulfonic acid (HEPES) buffer.

For the proliferation experiments, monocyte/macrophage, NK, and granulocyte subpopulations were identified using these criteria: monocytes/macrophages were identified as CD3^low^, CD11b^high^, CD4^low^, CD8^low^, CD19^low^, CD56^low^, CD14^high^, and SSC^low^. NK cells were identified as CD3^low^, CD11b^low^, CD4^low^, CD8^low^, CD19^low^, CD56^high^, CD14^low^, and SSC^low^. Granulocytes were characterized by the following profile: CD3^low^, CD11b^high^, CD4^low^, CD8^low^, CD19^low^, CD56^low^, CD14^low^, and SSC^high^. Cell counts were expressed as absolute numbers, based on collected beads (Precision Count Beads, ref: #424902, BioLegend, San Diego, CA, USA). For the monocyte/macrophage activation experiment, HLA-DR expression was analyzed within the population marked by CD3^low^, CD11b^high^, CD14^high^, and SSC^low^. In the NK and granulocyte activation experiment, CD69 expression was evaluated within the NK cells and the granulocyte populations, respectively characterized by the markers CD3^low^, CD11b^low^, CD4^low^, CD8^low^, CD19^low^, CD56^high^, and SSC^low^ and CD3^low^, CD11b^high^, CD4^low^, CD8^low^, CD19^low^, CD56^low^, and SSC^high^. Cells were labeled and assessed via flow cytometry on a BD FACS Canto II (BD Biosciences, Franklin Lakes, NJ, USA) in the configuration 4/2/2.

### 2.4. Analysis of the Cytokine Secretion in Human Peripheral Blood Mononuclear Cells

Peripheral blood mononuclear cells from *n* = 6 healthy donors were plated at a density of 330,000 cells per well. Incubation periods of either 24 or 48 h with either Veh. or 2LEID-N-9 were performed in order to evaluate the effects of the compounds on cytokine secretion, either in basal culture conditions or under CD3/CD28 stimulation (1 µg/mL and 2 µg/mL of anti-CD3 and anti-CD28, respectively). The supernatant was collected and frozen at −80 °C for subsequent cytokine analysis. The secretion of five cytokines was evaluated by an enzyme-linked immunosorbent assay (ELISA) (IL-2, IL-4, IL-13, IFN-γ, and TNF-α; ref: #740527, BioLegend).

### 2.5. Assessment of TNF-α Secretion in THP-1 Cells

THP-1 cells were sourced from ATCC (Manassas, VA, USA) and cultivated in RPMI 1440 medium supplemented with 10% fetal calf serum and antibiotics. A total of 500,000 cells per well were seeded into 24-well plates. The cells were pre-treated with either Veh. or 2LEID-N-9 at a sugar concentration of 11 mM for 30 min, followed by stimulation with LPS (1 μg/mL) at three different time points (16, 24, and 30 h). The supernatants (SNs) were collected and stored at −80 °C until needed. TNF-α levels were quantified using ELISA, following the manufacturer’s instructions (R&D Systems, Minneapolis, MN, USA).

### 2.6. Detection of the Active Substances Through High-Performance Liquid Chromatography Coupled with High-Resolution Mass Spectrometry

A *shotgun proteomics* approach based on liquid chromatography coupled to high-resolution tandem LC-HRMS/MS was employed to detect and identify bioactive compounds in 2LEID-N-9 with high sensitivity. Due to the complex nature of the matrix, protein recovery from the pharmaceutical formulation was required prior to the analysis.

Two capsules of 2LEID-N-9 were dissolved in 1 mL of LC-MS grade water (Merck Millipore, Darmstadt, Germany). To precipitate proteins from the sugar excipient, trichloroacetic acid (TCA) was added dropwise to reach a final concentration of 24%. The sample was incubated on ice for 4 h, followed by centrifugation at 13,000× *g* for 20 min at 4 °C. The resulting pellet was carefully collected and resuspended in Urea 6M Tris-HCl 50 mM, pH 8.

Tryptic peptides were generated from protein pellets via in-solution digestion using Trypsin Gold (Promega Corporation, Madison, WI, USA). The resulting peptides were purified by solid-phase extraction (SPE) using BondElute C18 cartridges (Agilent Technologies, Santa Clara, CA, USA). Elution was performed with 70% LC-MS grade acetonitrile (ACN); (Merck Millipore), and the eluates were fully evaporated overnight using a speed-vac concentrator. Dried peptides were reconstituted in water containing 0.1% formic acid (FA). Alongside the experimental samples, Veh. and blank controls (water + 0.1% formic acid) were processed and analyzed using the same LC–MS methodology and instrumental settings in order to verify the absence of background signals, contamination, or carryover.

In parallel, recombinant standards corresponding to each cytokine present in the 2LEID-N-9 formulation underwent the same digestion and purification protocol.

Peptide analysis was carried out using an Eksigent LC 425 chromatography system (AB SCIEX, Framingham, MA, USA) coupled to a TripleTOF 6600+ mass spectrometer (AB SCIEX). Chromatographic separation was performed under reversed-phase conditions at a flow rate of 5 µL/min, using a Luna Omega C18 capillary column (Phenomenex, Aschaffenburg, Germany). Mobile phase A consisted of water with 0.1% FA, and mobile phase B of ACN with 0.1% FA. Data acquisition was conducted in data-dependent acquisition (DDA) mode. The TOF-MS scan range was set from *m*/*z* 350 to 1250, and MS/MS scans were acquired in the range of *m*/*z* 100 to 1250. In parallel with sample analysis, instrument performance and system suitability were monitored using PepCalMix (AB SCIEX) and K562 cell lysate (AB SCIEX) as quality control standards.

Peptide identification and protein inference were performed using ProteinPilot software 5.0.2 (AB SCIEX), employing the Paragon algorithm. Carbamidomethylation of cysteine residues was selected as a fixed modification, and FASTA sequences of the target cytokines were used for database searching. False discovery rate (FDR) analysis was performed in parallel, with a 1% threshold for acceptable error.

Peptide identification results were exported in mzIdentML (.mzID) format and imported into Skyline (MacCos Lab Software V23.1.0.268) using the Import Peptide Search workflow to generate spectral libraries in .blib format. Independent libraries were constructed from the 2LEID-N-9 sample and from a mixture of digested recombinant-standard proteins. Skyline extracted precursor masses, fragment-ion intensities, and retention-time information to produce curated MS/MS reference spectra for all confidently identified peptides. A set of proteotypic peptides unique to the target proteins was selected for validation. For each peptide, experimental spectra from the sample library were directly compared against the corresponding spectra from the standard-protein library using Skyline’s spectral-matching metrics (dot-product similarity) and retention-time consistency. Concordance in fragment-ion patterns and chromatographic behavior between both libraries confirmed the correct identification of the peptides and validated the presence of the target proteins in the experimental sample.

### 2.7. Statistical Analysis

The methodology for the statistical analysis follows the recommendations of D.L. Vaux and J. Curtis et al. [19,20]. Accordingly, due to the small sample size (*n* < 5), inferential statistics were applied for data in Section 3.1, Section 3.2 and Section 3.4, only for indicative and exploratory purposes, as mentioned in the legends. Non-parametric Wilcoxon matched-pairs signed rank tests have been performed to analyze the data obtained from six donors (*n* = 6 independent values) (see Section 3.3), reporting the *p*-value and the standard error of the mean (S.E.M.). The graphs presented in all the figures were created using GraphPad Prism, Version 10.6.1.892 for Windows (GraphPad Software Inc., San Diego, CA, USA, updated on 23 September 2025).

## 3. Results

### 3.1. Exploratory Experiments Show That the Three Actives IL-12 (5 CH), IFN-γ (6 CH) and TNF-α (5 CH) of 2LEID-N-9 Slightly Enhanced Phagocytosis of Human Macrophages In Vitro and Modulated the Activation Status of Monocytes/Macrophages Under Basal Conditions in a Donor-Dependent Manner

**Figure 1:**
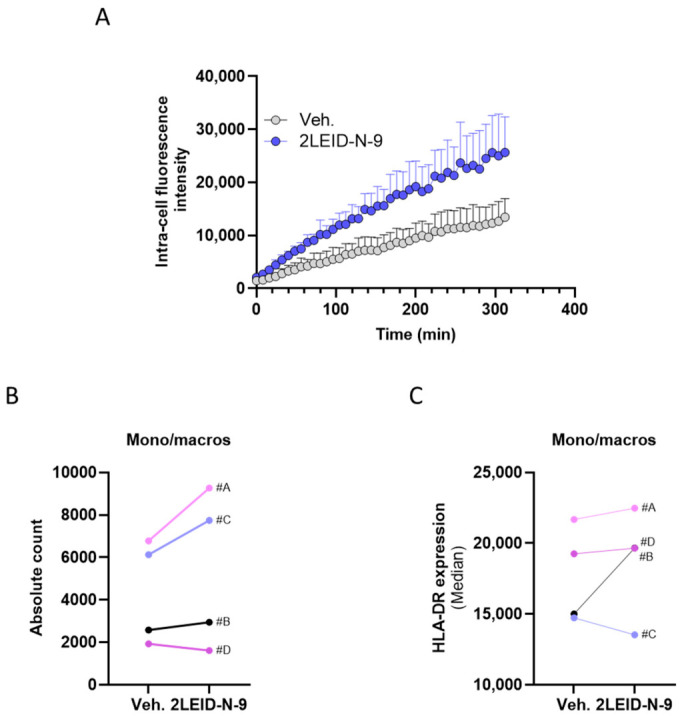
Effects of three actives from 2LEID-N-9—IL-12 (5 CH), IFN-γ (6 CH), and TNF-α (5 CH)—on the phagocytosis capabilities of monocyte-derived macrophages and monocytes/macrophages in vitro. (**A**) Monocytes isolated from one healthy donor were differentiated into macrophages for 6 days in the presence of GM-CSF and IFN-γ. The treatment with Veh. or 2LEID-N-9 started 24 h before inducing the phagocytosis. Each condition was performed in *n* = 6 replicates. Data are represented as the mean ± S.D. of the intracellular fluorescence intensity. For indicative and explorative purposes only, unpaired T test was performed at the final time point (*p* < 0.0001). (**B**,**C**) The three actives IL-12 (5 CH), IFN-γ (6 CH), and TNF-α (5 CH) from 2LEID-N-9 affected the cell number and the expression of HLA-DR of monocytes/macrophages in vitro, under basal conditions in a donor-dependent manner. Human peripheral blood mononuclear cells (PBMCs) from four healthy donors (#A, #B, #C and #D) were cultured for 48 h under standard culture conditions. The cells were immuno-stained and identified based on the marker expression detailed in the Materials and Methods Section on day 2 and analyzed by flow cytometry on day 3. (**B**) The monocyte/macrophage count, along with (**C**) their activation status (HLA-DR expression). Each data point (colored dot) represents the average of one triplicate per donor.

At first, the three actives, IL-12 (5 CH), IFN-γ (6 CH), and TNF-α (5 CH), employed in the 2LEID-N-9 capsule were evaluated in their capacity to affect macrophage functions. Macrophages play a crucial role in innate immunity by identifying and engulfing pathogens and debris, making them a pivotal target for examining phagocytic activity. The experimental setup included a control group treated with Veh. alone, to provide a baseline for comparison. The results presented in Figure 1, show that treatment with 2LEID-N-9 led to a time-dependent increase in phagocytosis compared to the Veh. control (Figure 1A). Over about 5 h, macrophages exposed to 2LEID-N-9 exhibited a tendency towards enhanced phagocytic response against the yeast *C. albicans*. Indeed, at the end of the incubation period the phagocytosis capabilities of the 2LEID-N-9-treated cells were nearly double of those of the Veh.-treated cells, according to the results from this experiment. These findings, while preliminary, suggest that 2LEID-N-9 may enhance the phagocytic activity of macrophages.

Then, the potential effects of 2LEID-N-9 were evaluated on human primary monocytes/macrophages derived from the blood from four donors (#A, #B, #C, and #D), focusing on cell proliferation and HLA-DR expression. HLA-DR, a critical component of the antigen presentation machinery, is a suitable marker of activation for monocytes [21]. This experiment was prompted by the known involvement of IL-12, IFN-γ, and TNF-α in monocyte/macrophage activation [22,23]. In particular, the experimental design involved the isolation of monocytes from the peripheral blood of four donors and their differentiation into macrophages. Upon treatment with 2LEID-N-9, a small enhancement in the mean cell proliferation was observed across macrophages from three donors out of the four tested (#A, #B, #C), indicating the compound’s potential to slightly promote macrophage growth in a basal state (Figure 1B), while being cautious as a donor’s inter-variability in their response occurred. Of note, the global tendency of the response for all the donors is shown in Supplementary Figure S1A. Again, this inter-variability was still observed in the markers’ expression analysis, as HLA-DR was upregulated by about 10% following 2LEID-N-9 treatment in donors #A, #B and #D (Figure 1C), while donor #C displayed a different response in comparison with the general trend because the increase in cell count was accompanied by a decrease in HLA-DR expression (Figure 1C and Supplementary Figure S1B). Donor #D was observed to respond with the highest increase in HLA-DR expression, and this was accompanied by a slight cell loss. Nonetheless, the overall small upregulation of both proliferation and HLA-DR expression in three out of the four donors suggests that 2LEID-N-9 may enhance phagocytic capacity, and exert immunomodulatory effects in monocytes/macrophages.

Overall, these preliminary results highlight 2LEID-N-9′s potential capability to modulate monocyte/macrophage function, mainly toward activation.

### 3.2. An Exploratory Experiment Shows That the Three Actives IL-12 (5 CH), IFN-γ (6 CH), and TNF-α (5 CH) from 2LEID-N-9 Displayed Small Immunomodulating Effects on Natural Killers and Granulocytes in Basal State, in a Donor-Dependent Manner

**Figure 2:**
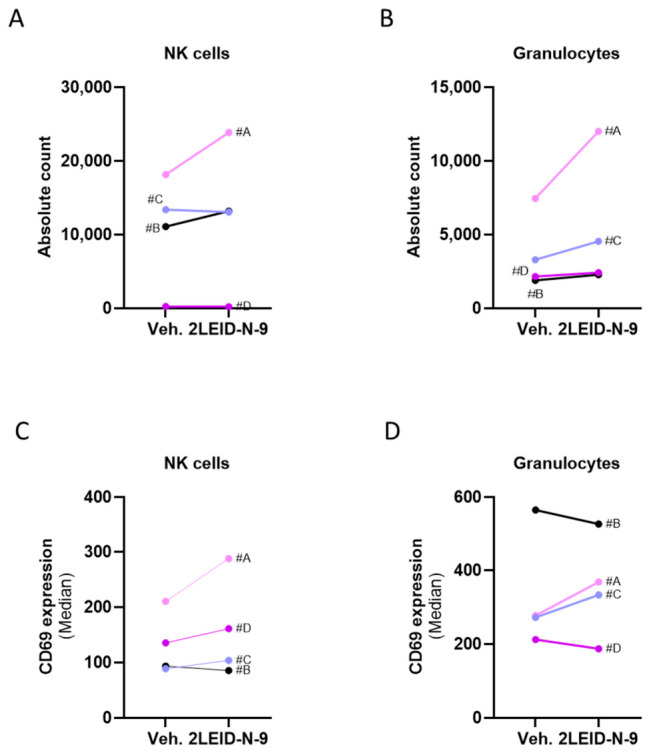
Donor-dependent effects of the three actives from 2LEID-N-9; IL-12 (5 CH), IFN-γ (6 CH) and TNF-α (5 CH) from 2LEID-N-9 on natural killers (NKs) and granulocytes in vitro. Human peripheral blood mononuclear cells (PBMCs) from four healthy donors (#A, #B, #C and #D) were cultured for 48 h in standard culture conditions. The cells were immuno-stained and identified based on the marker expression detailed in the Materials and Methods Section on day 2 and analyzed by flow cytometry on day 3. (**A**,**B**) The cell count along with (**C**,**D**) their activation status (CD69 expression) were evaluated. Each data point (colored dot) represents the average of one triplicate per donor.

The same analysis was pursued in the NK and granulocyte subpopulations, and cell count as well as the expression of the activation marker CD69 were chosen as indicators of the cell’s activation. We observed that 2LEID-N-9 slightly increased the proliferation of NK cells (Figure 2A), in donor #A and donor #B only, and granulocytes (Figure 2B), in all the tested donors, in basal culture conditions compared to Veh. The overall trend towards an increase is shown in Supplementary Figure S2A,B. Again, the inter-variability between donors was also reflected in the CD69 expression, which was increased in three out of the four tested donors in the NK cells (Figure 2C), and in two out of the four donors in granulocytes (Figure 2D). Overall, 2LEID-N-9 increased the expression levels of the activation marker CD69 by about 20% in comparison with Veh. in the basal state, in NK cells (Supplementary Figure S2C,D). However, due to donor-dependent variability and small effects, no conclusion could be drawn for granulocytes (Figure 2D and Supplementary Figure S2).

The overall body of data from this exploratory experiment indicates that the three actives from 2LEID-N-9 could have the potential to display small immune-modulating effects, which varies in terms of the magnitude and direction of the effect amongst the tested donors, on these cell subpopulations, in the assessed in vitro conditions. Keeping in mind the importance of inter-donor variability, further studies should be planned in samples from a larger cohort.

### 3.3. The Three Actives from 2LEID-N-9 (IL-12 (5 CH), IFN-γ (6 CH), and TNF-α (5 CH)) Enhanced Cytokine Secretion in Human PBMCs, in the Basal State, as Well as in a CD3/CD28-Stimulated Context

The exploration of 2LEID-N-9′s impact on cytokine secretion is a crucial part of the study. Building on the encouraging results reported in the previous sections, mainly focusing on innate immune cells, the research was expanded to determine how 2LEID-N-9 could affect cytokine profiles in human PBMCs derived from healthy donors. To strengthen and provide more robustness to the preliminary data obtained from the previous sections, the PBMCs in this experiment were retrieved from six donors.

**Figure 3:**
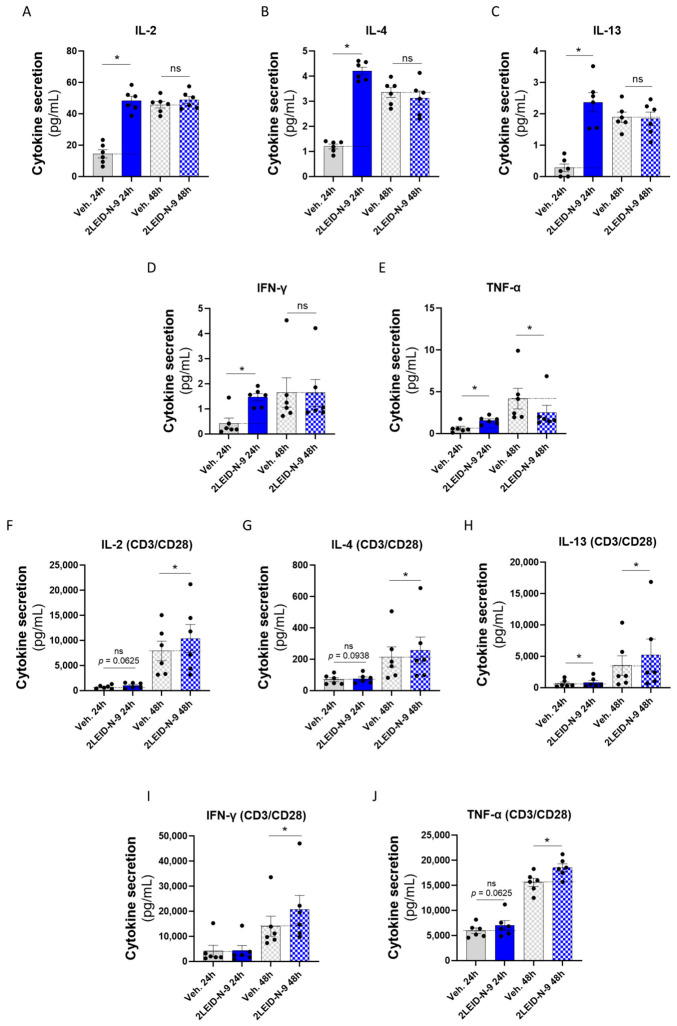
The three actives from 2LEID-N-9; IL-12 (5 CH), IFN-γ (6 CH), and TNF-α (5 CH) modulated the secretion of a panel of cytokines in PBMCs from healthy donors. PBMCs from *n* = 6 healthy donors were incubated with Veh. or with 2LEID-N-9 in standard culture conditions (**A–E**), or in the presence of CD3/CD28 antibodies (1 µg/mL and 2 µg/mL, respectively; **F–J**), and the secretion of five cytokines was assessed in the SNs by ELISA after either 24 or 48 h incubation periods. The secretion of IL-2, IL-4, IL-13, IFN-γ, and TNF-α was evaluated. Data are shown as mean ± S.E.M. of the cytokine secretion in pg/mL. Each dot represents the mean measure of one duplicate per donor. Dotted lines are drawn to highlight the effect of 2LEID-N-9 in comparison with Veh. Non-parametric Wilcoxon matched-pairs signed-rank tests have been performed to analyze the data (**A–J**): * *p* < 0.05; ns: non-significant.

Cells were either cultivated in a basal state or stimulated with CD3/CD28 to mimic the T-cell receptor engagement. Indeed, it has been reported that the chosen CD3/CD28 stimulatory condition could partially mimic a stimulation induced by antigen-presenting cells, thereby promoting the activation of the cells of adaptive immunity [24]. Briefly, PBMCs were incubated with Veh. or 2LEID-N-9, in basal culture conditions (Figure 3A–E), or in the presence of CD3/CD28 antibodies (Figure 3F–J). The secretion of five cytokines (IL-2, IL-4, IL-13, IFN-γ, and TNF-α) was assessed in the SNs by the ELISA method, after 24 and 48 h incubation periods. In the basal state, statistically significant increases in all the above-mentioned cytokines were observed after 24 h of treatment with 2LEID-N-9 compared to Veh. For instance, IL-2 secretion increased by 3.3 times (Figure 3A); IL-4 secretion by 3.5 times (Figure 3B); IL-13 secretion by 8.25 times (Figure 3C); IFN-γ secretion by 3.4 times (Figure 3D); and TNF-α secretion by 2.4 times (Figure 3E). No significant change was obtained at 48 h, except for a 1.6-fold decrease in TNF-α secretion (Figure 3E). Interestingly, in CD3/CD28 stimulation conditions, although the increasing tendencies did not reach significance at 24 h (except for IL-13), all cytokines showed significant increases at 48 h: IL-2 by 1.3-fold (Figure 3F); IL-4 by 1.1-fold (Figure 3G); IL-13 and IFN-γ by 1.4-fold (Figure 3H,I); and TNF-α by 1.2-fold (Figure 3J). In addition, the secretion of five other cytokines, TNF-β, B-cell activating factor (BAFF), sCD40L, IL-3, and GM-CSF, was evaluated in CD3/CD28 stimulation conditions. This body of data supports an overall effect of 2LEID-N-9 in slightly stimulating cytokine secretion, as all of them were increased compared to Veh., after both of the 24 or 48 h incubation periods tested (Supplementary Figure S3).

These preliminary results are consistent and, as some of them reach statistical significance, they suggest that 2LEID-N-9 may act as an immuno-enhancing agent, able to slightly up-regulate the secretion of cytokines in both resting and stimulated conditions. In a basal state, the significant peak in cytokines at 24 h suggests a bona fide response of the immune machinery. The observation that this effect subsides at 48 h, in the absence of secondary stimuli, suggests a time-dependent effect of the tested capsule. However, a proper kinetics experiment incorporating additional time points would be necessary to confirm this conclusion. Moreover, under CD3/CD28 stimulation, the effect seems to only emerge at 48 h (except for IL-13, see Figure 3H), characterized by a slight pleiotropic increase in the mitogenic cytokine IL-2, in Th1 response cytokines (IFN-γ and TNF-α) and in Th2 response cytokines (IL-4, and IL-13). Since no significant change was observed in the absolute counts of PBMC subpopulations, this global cytokine boost, though small, could imply an enhancement of the cellular secretory capacity rather than clonal expansion. Further studies are ultimately needed to validate this hypothesis.

### 3.4. The Three Actives from 2LEID-N-9 (IL-12 (5 CH), IFN-γ (6 CH), and TNF-α (5 CH)) Showed a Trend Towards Increased TNF-α Secretion in Lipopolysaccharide-Stimulated THP-1

**Figure 4:**
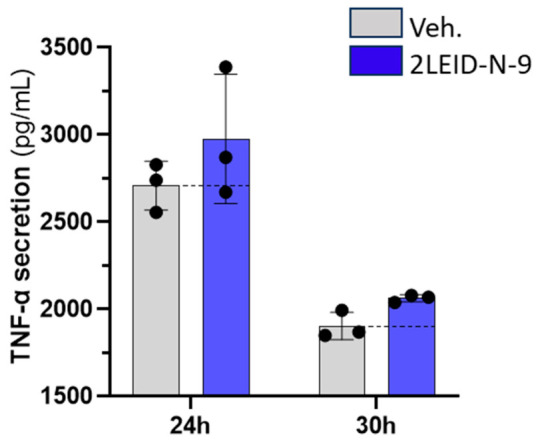
The three actives from 2LEID-N-9; IL-12 (5 CH), IFN-γ (6 CH), and TNF-α (5 CH) tended to slightly increase the secretion of TNF-α in the human monocytic cell line THP-1 under LPS-stimulated conditions. THP-1 cells were pre-treated with either Veh. (gray histograms) or 2LEID-N-9 (blue histograms) for 30 min, followed by stimulation with 1 μg/mL LPS. The cells were treated for a total duration of 24 and 30 h. The supernatants were collected and stored at −80 °C until needed. The levels of TNF-α were quantified using enzyme-linked immunosorbent assay (ELISA). Data are derived from one experiment and are shown as the mean ± S.D. of cytokine secretion (in pg/mL), obtained for *n* = 3 replicates at the two time points, 24 and 30 h. Dotted lines are drawn to highlight the effect of 2LEID-N-9 in comparison with Veh. For indicative and explorative purposes only, multiple unpaired T tests were performed (24 h: *p* = 0.33; 48 h: *p* = 0.063).

Tumor necrosis factor (TNF)-α is a crucial pro-inflammatory cytokine involved in the body’s defense against pathogens. It is produced by activated immune cells such as macrophages and T-cells and initiates a robust inflammatory response to clear infections. As TNF-α enhances phagocytosis, activates immune cells, induces the production of other cytokines, promotes apoptosis of infected cells, increases antimicrobial activity, and supports the adaptive immune response by facilitating dendritic cell maturation, the last part of this study sought to evaluate the effect of 2LEID-N-9 on TNF-α secretion. Regarding its similarity to human macrophages, the human monocytic cell line THP-1 is commonly used in screening experiments for evaluating immune activators or inhibitors [25]. Thus, in our study, TNF-α secretion was measured in stimulated THP-1 cells to evaluate if the results from the previous section could be reproduced in another cellular model (Figure 3J). As an activation control, lipopolysaccharide (LPS) was employed to treat the cells, and a time-dependent evaluation of TNF-α release was performed after 16, 24, and 30 h through ELISA. The results of LPS alone are shown in Supplementary Figure S4A, confirming that the cells were potent TNF-α secretors, at each of the assessed time points. In parallel, the cells were treated with either Veh. or 2LEID-N-9 under LPS-stimulated conditions (Figure 4). Interestingly, a tendency towards a small increase in the secretion of TNF-α of about 10% was noticed after both 24 and 30 h, in the 2LEID-N-9-treated cells in comparison with the Veh.-treated ones. Of note, it seemed that 2LEID-N-9 could sustain a prolonged TNF-α secretion over time compared to that induced by bacterial threat, as the TNF-α measures were still more elevated in the 2LEID-N-9 treatment conditions than in the LPS ones, after either 24 or 30 h (Supplementary Figure S4B).

In conclusion, this body of data, while preliminary, indicates a trend towards a sustained TNF-α secretion effect elicited by 2LEID-N-9 in THP-1 cells after 24 and 30 h, underscoring, once again, the potential effect of the tested actives in slightly enhancing the secretory capacity of immune cells, under stimulated conditions. Further studies are ultimately needed to explore the potential of the tested item in the context of bacterial infection.

### 3.5. IL-12, IFN-γ, and TNF-α Were Detected Within the 2LEID-N-9-Diluted Pillules Assessed by Liquid Chromatography Coupled with High-Resolution Mass Spectrometry

To confirm the presence of target proteins in 2LEID-N-9, data-dependent acquisition (DDA) datasets were acquired by LC-HRMS/MS, and the results obtained with ProteinPilot were used as the primary peptide/protein identification source. Peptide analysis revealed 14 peptide sequences identified at 1% FDR, matching the target three proteins, thus allowing their identification.

To validate the protein identifications, spectral libraries were independently generated in Skyline for the 2LEID-N-9 digest and for a mixture of recombinant standards, each imported as an mzIdentML (.mzID) file. Both libraries contained curated MS/MS spectra including precursor *m*/*z*, fragment-ion intensities, and retention-time information. Skyline’s library-matching tools were then used to compare all peptides detected in the 2LEID-N-9 library against the corresponding entries in the standard-protein library. Peptides showing spectral concordance—defined by consistent fragment-ion patterns, high dot-product similarity, and alignment of retention times—were considered confidently validated. Using this unbiased spectrum-to-spectrum comparison, every peptide associated with IFN-γ, IL-12 (α and β subunits), and TNF-α that was present in the sample library also matched the recombinant-standard library, confirming the presence of all target proteins in 2LEID-N-9 even at extremely low abundance.

All peptides that achieved correct spectral matching between the 2LEID-N-9 library and the recombinant-standard library are listed in Table 1. For all target proteins except one, at least two peptides showed high spectral similarity and consistent retention-time alignment, providing robust confirmation of their presence. For the remaining protein, a single peptide fulfilled the matching criteria; however, its spectral agreement with the standard was unambiguous, supporting its confident identification.

Several of the matched peptides contained carbamidomethylated cysteine residues, a modification introduced exclusively during the sample-processing alkylation step. The presence of this modification in both the sample and standard-derived spectra provides additional confirmation that these peptides originated from in-sample tryptic digestion, further reinforcing the validity of their identification.

**Figure 5:**
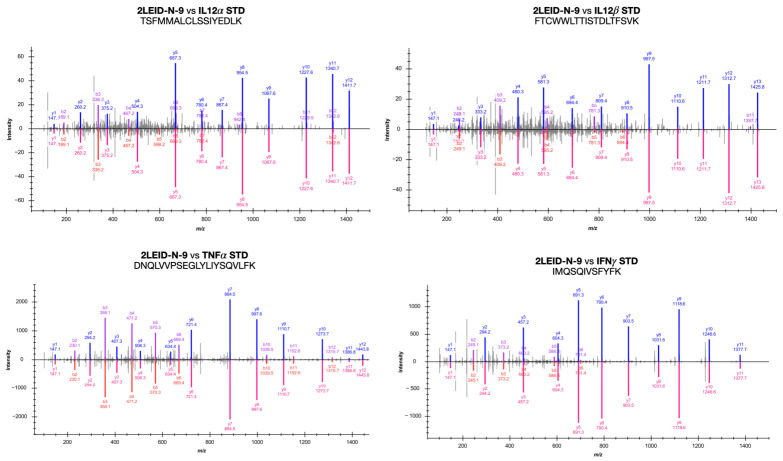
Mirror spectral comparison between the 2LEID-N-9 sample (blue signals) and the recombinant-standard library (red signals) for one validated peptide. Dot-product (dotp) similarity values were calculated using the fragment ions shared between both spectra, ensuring that the score reflects genuine concordance in the MS/MS fragmentation pattern. The overlap in major fragment-ion intensities supports correct peptide identification. Chromatograms for all validated peptides are provided in the Supplementary Materials (Figures S5–S15).

A representative example of the spectral-matching validation is shown in Figure 5, illustrating the mirror plot between the 2LEID-N-9 library and the corresponding recombinant-standard spectrum for one of the validated peptides. In the mirror plot, fragment-ion intensities are normalized by the Skyline library-matching algorithm, which scales both spectra to their respective base peaks to enable direct visual comparison.

The high concordance in fragment-ion intensities and overall spectral profiles exemplifies the agreement observed across the dataset. Complete mirror plots for all peptides listed in Table 1, together with their corresponding extracted ion chromatograms, as well as negative controls graphs for LC-HRMS analysis are provided in Supplementary Figures S5–S23. These control samples revealed only baseline noise, with no detectable chromatographic peaks or protein-related signals. This unequivocally confirms that the protein-specific signals obtained were exclusively derived from the experimental samples, validating that the results are not attributable to the background contamination, carryover effects, or artifactual signals.

**Figure 6:**
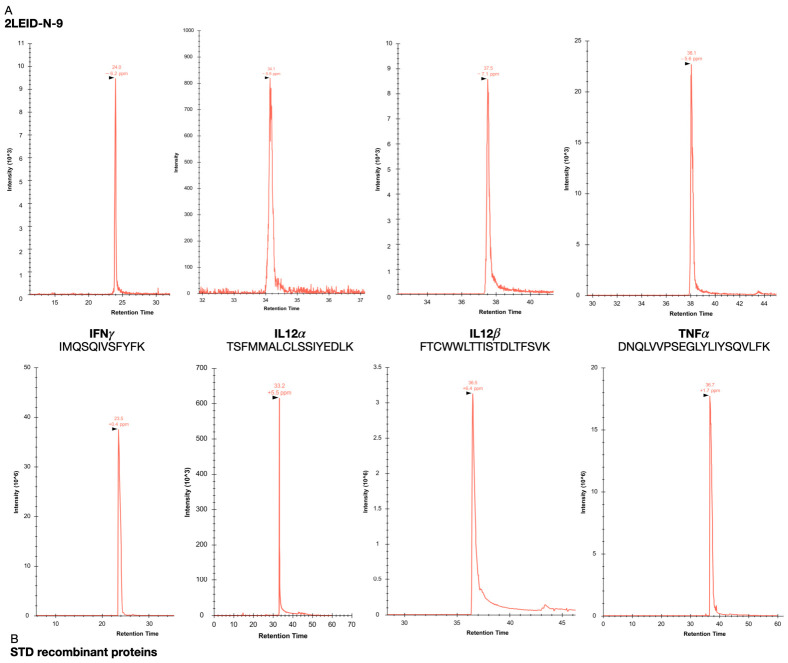
Extracted ion chromatogram of one selected peptide per protein in 2LEID-N-9 (**A**) and standard (STD) recombinant protein (**B**) from each target protein.

Extracted ion chromatograms (XICs) for the peptides indicated in Figure 5 were compared between 2LEID-N-9 and the recombinant standard (Figure 6). In all cases, the precursor *m*/*z* values matched exactly within instrument tolerance (±10 ppm), confirming peptide identity. Retention times (RTs) showed minor shifts between the two matrices, likely due to matrix effects in 2LEID-N-9, which contained extremely low protein levels. Despite these shifts, co-elution patterns were consistent (0.5–1 min) and the expected monoisotopic peaks were observed in both datasets. In the experimental sample, isotopic distributions were dominated by the monoisotopic peak.

## 4. Discussion

In their study entitled “global epidemiological trends in the incidence and deaths of acute respiratory infections from 1990 to 2021”, Chen et al. reported that endemic respiratory diseases caused 2.5 million deaths in 2019 [26], and such infections are still a major health problem worldwide. As a consequence, the management of respiratory diseases has gained significant attention in recent years. Traditional therapeutic approaches often focus on symptom alleviation and long-term disease control but can be associated with adverse effects or limited efficacy in certain populations [27]. Consequently, there is an emerging interest in complementary and alternative therapeutic strategies that target the underlying immunological pathways. Micro-immunotherapy represents an interesting approach in this context, employing formulations mainly based on cytokines at LD and ULD to modulate immune responses. In particular, the current study explored the effects of the combination of the three actives (IL-12 (5 CH), IFN-γ (6 CH), and TNF-α (5 CH)) from MIM 2LEID-N in several populations of immune cells. Being the ninth capsule of the medicine, the tested item is called 2LEID-N-9 throughout the manuscript. In the subsequent section, we discuss the preliminary data presented here, and delve into the intricate roles of various immune cell types within the context of ENT infections. In the discussion of our manuscript, it is acknowledged that there exists a notable gap between preclinical and clinical research. Our results are approached with a focus on “in vitro to patient” translation. However, caution is exercised in our conclusions due to a keen awareness of the limitations inherent in our study, which are also addressed later in the paper. This careful consideration underscores, once again, the preliminary nature of our findings and the need for further validation in subsequent research phases.

The opening segment of this discussion focuses on innate immunity, examining the results obtained in exploratory experiments aimed at unveiling potential effects of 2LEID-N-9 on primary immune cells. Innate immune cells, such as neutrophils, granulocytes, macrophages, and NK cells, provide the first line of defense against pathogens encountered in ENT infections. Their rapid response and ability to recognize common bacterial and viral components are crucial for controlling infection and initiating further immune responses. The second part of this discussion refers to the 2LEID-N-9 effects observed in PBMCs and the changes induced by this product on the secretion of several cytokines, including TNF-α. This section provides explanations on how 2LEID-N-9 could also affect adaptive immunity.

At first, the results of the experiment performed in human macrophages showed a trend towards an increase in phagocytic capabilities by a 1.9-fold change after about 5 h compared to the Veh.-treated control (Figure 1A). This time-dependent enhancement of phagocytosis, previously reported in another research about the MI product 2LEID [17], suggests the potential of 2LEID-N-9 to modulate macrophage activity and strengthen innate immune responses. Macrophages are vital for clearing pathogens and debris, and potentially supporting their functions could be particularly beneficial in the context of infections such as those affecting the respiratory tract, where efficient clearance is crucial [28]. The observed effects could be attributable to the possible synergistic action of the three actives from 2LEID-N-9: IL-12 (5 CH), IFN-γ (6 CH), and TNF-α (5 CH). Indeed, TNF-α and IFN-γ were both reported to be potent inducers of phagocytosis in macrophages [29], especially when they were associated together, due to the induction of autocrine positive feedback loops [30,31]. Regarding the possible role of IL-12 on the phagocytic capabilities of macrophages, a study showed that IL-12 had the potent ability to activate macrophages during intracellular infections, primarily by stimulating the release of IFN-γ from these cells [32]. According to this study, IL-12 has an indirect effect on phagocytosis, mediated by the upregulation of IFN-γ. Thereby, the three cytokines together can synergistically activate macrophage functions.

The next sections of results are aimed at underscoring the effects of 2LEID-N-9 on monocytes and in monocyte-derived macrophages when cultured in basal/unstimulated conditions. Monocytes and their derivative macrophages play a crucial role in the immune system, particularly in the context of respiratory tract infections [33]. During infections, monocytes and macrophages act as first responders, engulfing pathogens through phagocytosis and initiating the immune response by presenting antigens to T-cells. Antigen presentation could be considered at the interface between immune and adaptive immunity, and the clearance of the infection depends on its efficiency. Of note, while HLA class I molecules are fundamental for antigen presentation to cytotoxic T-cells, HLA-DR plays a role in presenting antigens to helper T-cells [34]. Thus, the observed trend towards the enhancement in HLA-DR expression following 2LEID-N-9 treatment at basal levels in three out of the four donors, especially in donor D#, is of particular interest (Figure 1C). It is, however, important to be careful with these observations, as, in our experiment, one donor out of the four tested responded in an opposite direction, reflecting the existence of an inter-donor variability, which adds another limitation in the conclusions that can be drawn in this study. When working with human primary cells, it is normal to encounter low responders, probably due to exhausted cells, along with robust and sensitive responders to a specific stimulus. For that reason, further studies on a larger cohort are necessary before drawing any conclusions. In the meantime, the exploratory results still indicate potential effects that merits attention.

It is worth mentioning that diminished HLA-DR expression and decreased IL-10 production were observed, in vitro, during severe respiratory syncytial virus infections, suggesting that a disrupted innate immune response could be a key factor contributing to the severity of the disease [35]. Thus, if applied in the context of respiratory infections, the upregulation of HLA-DR on macrophages could improve antigen presentation efficiency, speeding up the T-cell activation process and leading to a stronger immune response. Future studies are needed in this specific axis of research to investigate the potential of this MI formulation in supporting macrophage’s activation and function.

Regarding the investigations in NK cells and granulocytes, cell counts and the expression of the activation marker CD69 were chosen to evaluate the cell activation status. CD69 was found to be required for the activated-NK-cell-mediated killing of resistant targets [36]. The results showed that 2LEID-N-9 could slightly affect the proliferation of these two innate immune cells (Figure 2A,B) and the expression of the tested marker, at basal level, in a donor-dependent manner (Figure 2C). These preliminary in vitro results provide the first pieces of evidence on the immunomodulatory effect of the three combined actives from 2LEID-N-9 on these cells and highlight the need for future studies that should take into account donor variability. This body of data suggests that a larger cohort of samples is warranted to further investigate the potential of the tested MI formulation in modulating immune responses.

In the meantime, the overall tendency observed in NK cells could have a possible explanation: as NK cells are reported to express IL-12 receptors IL-12Rβ1 (low affinity) and β2 (high affinity), the presence of LD of IL-12 in the tested item could have played a role in the increased proliferation and activation observed in these cells (Figure 2A,C) [37]. Further studies are ultimately needed to validate this hypothesis, which can unveil the possible interaction of LD-based actives with cytokine receptors.

We then focused our attention on the possible effects of 2LEID-N-9 on cytokine secretion profiles in both unstimulated, or resting cells, and activated human PBMCs. Our observations thus highlighted that 2LEID-N-9 significantly enhanced the secretion of several cytokines, including IL-2, IL-4, IL-13, and IFN-γ, and TNF-α, either in basal culture conditions or following T-cell activation, mimicked by CD3/CD28 receptor engagement (Figure 3). The global cytokine secretion profile reveals a temporality of the effects that differs between basal and stimulated conditions. Under basal state, the effect is significant at 24 h, which then subsides at 48 h, suggesting its transient nature, without secondary stimuli. Only TNF-α shows a different behavior compared to the other cytokines tested (Figure 3E) because at 48 h its levels significantly decreased. This small downregulatory effect, only observed under basal conditions, could possibly involve negative feedbacks or the induction of anti-inflammatory mediators in a late stage, not investigated in this study. However, under CD3/CD28 stimulation, the increase in the tested cytokine secretion, including TNF-α (Figure 3F–J), only emerges at 48 h and not at 24 h, suggesting that the kinetics of activation differs depending on the “activation background” of the cells. Nonetheless, the increase in mitogenic cytokine IL-2 in Th1 response cytokines (IFN-γ and TNF-α) and in Th2 response cytokines (IL-4, and IL-13) seems to support the hypothesis that 2LEID-N-9 can sustain the secretory panel of cytokines involved in both Th1 and Th2 responses.

The results observed on these particular cytokines could possibly be explained by the composition of 2LEID-N-9 itself, as IL-12, IFN-γ, and TNF-α were previously reported to trigger the secretion of the five cytokines analyzed, even if, to the best of our knowledge, none of such studies were done on this LD-based MIM.

For instance, IL-12p70, IL-12p40 homodimer, and IL-12p40 monomer were shown to stimulate TNF-α production in mouse peritoneal macrophages [38]. Additionally, IL-12R signaling triggers the activation of mitogen-activated protein kinase kinase 3/6 (MKK) and p38 MAPK, facilitating the secretion of IFN-γ in activated T-cells and Th1 cells [39]. Studies have also demonstrated that pre-treating CD4^+^ and CD8^+^ T-cells with IL-12 boosts the production of TCR-induced IFN-γ, TNF-α, IL-13, and IL-4, while also enhancing oxidative metabolism [40,41]. Evidence that IL-12 could amplify the immune response by boosting IL-4 production was also demonstrated in invariant NKT cells [42]. Regarding the effect of IFN-γ on such cytokines, Bocek et al. reported that the incorporation of IFN-γ into the priming cultures of C57BL/6 IFNγ^−/−^ CD4^+^ T-cells increased their production of IL-4, as well as IL-13, but did not impact that of TNF-α [43]. In a model of murine peritoneal macrophages, while IFN-γ did not induce expression of TNF mRNA, it still enhanced the cytoplasmic accumulation of TNF mRNA induced by LPS [44]. The positive effect of IFN-γ on direct TNF-α secretion was still documented in a model of human keratinocytes in which the smallest tested concentration of 50 ng/mL of IFN-γ significantly induced 8 pg/mL of TNF-α [45]. Even if, to the best of our knowledge, there is no study documenting the direct effect of IFN-γ in inducing the secretion of IL-2, several studies reported that IFN-γ could induce the expression of IL-2R α chain (IL-2Rα) subunit at the transcriptional level [46,47,48], as well as that of the IL-2Rγ chain [49]. Finally, regarding TNF-α, this factor has been shown to induce the production of IL-13 in human group 2 innate lymphoid cells [50].

As one of the major effects of 2LEID-N-9 was observed on IL-2, it is worth discussing in more detail the implication of this cytokine within the context of ENT. Regarding the pleiotropic role of IL-2, as nicely summarized by Bendickova et al. [51], the observed elevations in its secretion indicate a possible enhancement in the orchestration of both innate and adaptive immunity. Indeed, IL-2 is a pivotal cytokine in the regulation and modulation of the immune system. Its role in hematopoiesis and immune cell proliferation has been extensively studied, as it enhances the survival and proliferation of activated T-cells and various myeloid cell types through signal transduction pathways, which include tyrosine phosphorylation of protein substrates that are shared by both lymphoid and myeloid lineages, such as pp200, pp180, pp92, and pp42 [52].

After observing the trend towards an increase in TNF-α within a model of PBMCs in both the basal/unstimulated state and CD3/CD28-stimulated state, our focus shifts towards other cellular systems to gain a deeper understanding of the underlying mechanisms of this inflammatory cytokine. Thus, this pilot study was finally enriched by the analysis of the effect of 2LEID-N-9 on TNF-α secretion in another model of macrophages, the THP-1 cells, in the context of LPS-activated immune response. This transition to LPS stimulation conditions provides preliminary data on the effects of the tested item in a bacterial-induced inflammatory model.

Macrophages serve crucial roles in innate immunity, not only due to their phagocytic abilities, as highlighted earlier, but also as significant producers of pro-inflammatory cytokines and antigen-presenting cells [53]. Tumor necrosis factor -α is known for its role in orchestrating the immune defense against pathogens, particularly in the respiratory tract [54]. Elevated TNF-α levels are often associated with heightened immune responses necessary during respiratory tract infections [55], as they enhance the phagocytosis of pathogens, recruit other immune cells to the site of infection, and play a crucial role in cytokine cascades leading to effective pathogen clearance. The use of the human monocytic cell line THP-1 as a model in this study is pertinent, given its similarity to human macrophages [25]. THP-1 cells, derived from a leukemia patient, have been extensively used in scientific research to evaluate immune modulators because they efficiently differentiate into macrophage-like cells upon stimulation. This ability makes them an excellent model for studying macrophage function and cytokine production, closely mirroring the human physiological response to infections. In our experimental setting, THP-1 cells treated with 2LEID-N-9 responded by a slight increase in TNF-α secretion after 24 and 30 h in comparison with the Veh.-treated cells (Figure 4), suggesting that 2LEID-N-9 might enhance immune activation in this bacterial inflammatory model. This temporal aspect of cytokine release is particularly relevant, as it could reflect the physiological response to persistent infections, like those seen in chronic or severe respiratory tract infections. Typically, respiratory infections elicit strong TNF-α responses as part of the innate immune system’s first line of defense [55], aiming to contain and eliminate pathogens efficiently. In our experimental setting, as the cells were stimulated with LPS, the upregulation of TNF-α secretion in the 2LEID-N-9-treated cells thus highlighted its potential application as an adjuvant treatment in bacterial infections of the respiratory tract. By potentially modulating macrophage activity towards a more robust pro-inflammatory profile, 2LEID-N-9 could help in managing infections where enhanced immune response is necessary. However, caution must be exercised, as while TNF-α plays a crucial role during the initial phase of the immune response, it can worsen the condition during the later effector phase [56], underscoring the need for finely tuned therapeutic strategies. Overall, further research is essential to elucidate the detailed mechanisms by which 2LEID-N-9 influences TNF-α secretion and the secretion of other cytokines in human macrophages (Figure 3 and Figure 4), and its effects on other immune pathways.

Finally, the application of LC-HRMS/MS in this study successfully confirmed the presence of the active ingredients IL-12, IFN-γ, and TNF-α within the assessed capsule of 2LEID-N-9 (Figure 5). The high sensitivity and specificity of LC-HRMS/MS were critical for accurately identifying these low-abundance biomolecules within the complex sugar-matrix. These findings have a big impact because they validate the formulation of the three actives and establish the first foundation proof that homeopathically prepared cytokines in MIM are present within the MI product.

Overall, these preliminary findings must be considered as exploratory in vitro evidence highlighting the potential capability of the three actives from the MIM 2LEID-N-9 to exert small immunomodulatory effects in vitro, across both the innate and adaptive immune cell model. For the potential mechanisms of the observed trends, we believe that the cytokines used at LD in the 2LEID-N-9 formulation might engage cytokine receptors with high affinities, initiating subtle immune responses. For example, even the low-level activation of IL-12 receptors could trigger downstream signaling cascades, such as the activation of the STAT pathway in T-cells, which enhances IFN-γ production and potentiates Th1 responses. Similarly, TNF-α could activate NF-κB pathways, promoting inflammatory responses and enhancing phagocytic activity. This body of data open avenues for further, more detailed mechanistic and validation studies aimed at exploring the potential of MI formulations in the management of infections, such as those affecting the ENT. It is important to acknowledge several limitations that necessitate further investigation. First of all, a single concentration of the MI formulation was tested in this study. While a dose–response curve is important to establish the relationship between the concentration and the effect, the sugar carrier of MIM is a biological constraint, as previously discussed [18], due to the fact that mammalian cells are not able to hydrolyze sugar molecules such as sucrose, which are then accumulated in the cytoplasm inducing autophagy and osmotic stress. Then, the relatively small sample size utilized in this study, especially in Section 3.1, Section 3.2 and Section 3.5 limits the generalization of the results. To ensure robust statistical validation, future studies should endeavor to confirm these results using larger sample sizes. We are currently conducting further research to explore these mechanisms in depth, which includes expanding the panel of tested cytokines and exploring the activation of signaling pathways, such as examining the phosphorylation status of the NF-κB and STAT pathways, for instance, to provide deeper insight into the molecular mechanisms at play. Additionally, potential next steps involve investigating the long-term effects of 2LEID-N-9 on immune cells, and exploring its interaction with other therapeutic agents commonly used in the treatment of ENT. Therefore, in the future, we also consider employing in vivo models of viral or bacterial infections, to evaluate if the immunomodulatory effects induced by 2LEID-N-9 translate into clinical benefits in these contexts. Such studies will help frame the scope and impact of our work more comprehensively.

## 5. Conclusions

The findings of this pilot study reveal, for the first time, promising insights into the potential effects of 2LEID-N-9. The results indicate that the tested items displayed immunomodulatory effects and trends in vitro in a panel of several immune models, including human PBMCs, primary macrophages, and THP-1 cell line. This body of data opens avenues for further investigations aimed at exploring the potential of MI formulations, in supporting immune response in the context of such as infections, including those affecting the ENT. However, it is important to acknowledge several limitations, such as the small sample size and donor inter-variability, which limit the generalization of the results. Future studies with larger sample sizes, a larger panel of immune markers, cytokine quantification, and in vivo investigations are ultimately needed to validate these initial findings and fully elucidate the therapeutic potential of 2LEID-N-9.

While these findings are preliminary, they lay the groundwork for future mechanistic investigation. The analytical detection of the actives by LC-HRMS/MS and the observed biological trends also establish a material and functional proof of concept for continued MIM research.

## Figures and Tables

**Table 1:** Peptides showing successful spectral matching between the 2LEID-N-9 sample and recombinant-standard spectral libraries.

Accessions	Start Position	Best Conf	Sequence	Modifications	Obs *m*/*z*	Theor *m*/*z*	Dotp to Standard
sp|P29459|IL12A_HUMAN	152	99	QIFLDQNMLAVIDELMQALNFNSETVPQK		1117.2310	1117.2316	0.86
sp|P29459|IL12A_HUMAN	116	99	TSFMMALCLSSIYEDLK	Carbamidomethyl	1004.9751	1004.9754	0.87
sp|P29460|IL12B_HUMAN	140	99	FTCWWLTTISTDLTFSVK	Carbamidomethyl	1103.5480	1103.5481	0.82
sp|P29460|IL12B_HUMAN	251	99	QVEVSWEYPDTWSTPHSYFSLTFCVQVQGK	Carbamidomethyl	1202.5620	1202.5609	0.95
sp|P01375|TNFA_HUMAN	121	99	DNQLVVPSEGLYLIYSQVLFK		809.1060	809.1052	0.94
sp|P01375|TNFA_HUMAN	208	99	LSAEINRPDYLDFAESGQVYFGIIAL		967.8273	967.8271	0.91
sp|P01579|IFNG_HUMAN	67	99	IMQSQIVSFYFK		745.8885	745.8892	0.95

## Data Availability

The data of the current study are available from the corresponding author upon reasonable request.

## Supplementary Materials

The following supporting information can be downloaded at: https://www.mdpi.com/article/10.3390/cimb48060566/s1.

## Abbreviations

ACN: acetonitrile; BAFF: B-cell activating factor; CH: centesimal Hahnemannian dilutions; DDA: data-dependent acquisition; DOTP: DOT-product; EFS: Etablissement Français du Sang, ELISA: enzyme-linked immunosorbent assay; ENT: ear, nose, and throat; FA: formic acid; FDR: false discovery rate; GM-CSF: granulocyte macrophage colony-stimulating factor; HEPES: 4-(2-hydroxyethyl)-1-piperazineethanesulfonic acid; HLA: human leukocyte antigen; hr: human recombinant; IFN: interferon; IL: interleukin; LD: low dose; LC-HRMS/MS: liquid chromatography coupled high-resolution mass spectrometry; LPS: lipopolysaccharide; MFI: median of fluorescence intensity; MI: micro-immunotherapy; MIM: micro-immunotherapy medicines; MKK: mitogen-activated protein kinase kinase; NK: natural killer cells; PBS: phosphate-buffered saline; PBMCs: peripheral blood mononuclear cells; RPMI: Roswell Park Memorial Institute; S.D.: standard deviations; S.E.M.: standard error of the mean; SNs: supernatants; SPE: solid-phase extraction; STD: standard; TCA: trichloroacetic acid; Th1: helper CD4^+^ T-cells type 1; TNF: tumor necrosis factor; ULD: ultra-low doses; Veh.: vehicle.
