# Supplementary material for: In Vitro Effects of the Three Actives IL-12 (5 CH), IFN-γ (6 CH), and TNF-α (5 CH) from the Micro-Immunotherapy Medicine 2LEID-N® on Several Populations of Immune Cells
Source: Curr Issues Mol Biol. 2026 May 28;48(6):566. doi: 10.3390/cimb48060566 (PMC13298170; doi:10.3390/cimb48060566)
Supplement: Supplementary file 1 [file cimb-48-00566-s001.zip › cimb-4240628-supplementary.pdf]

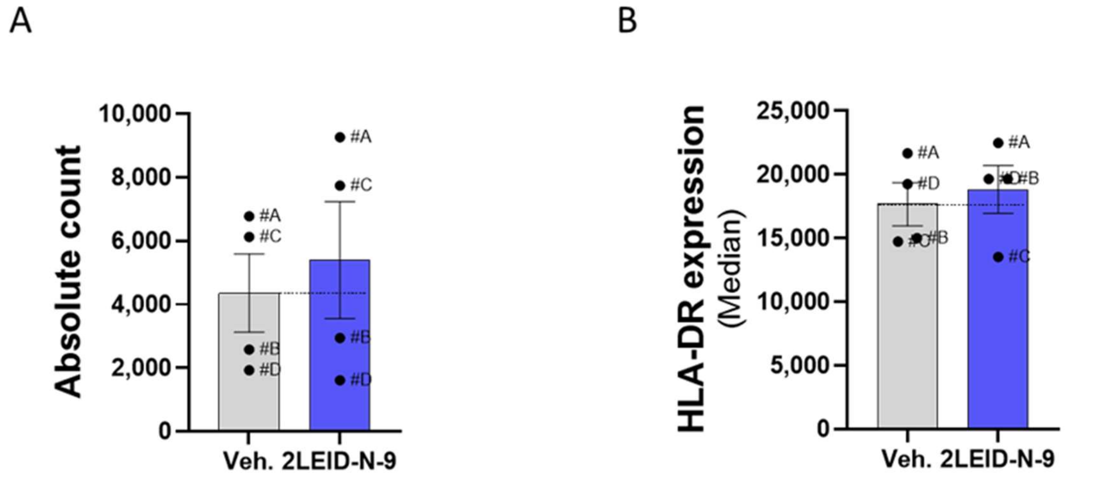

Supplementary Figure S1. Human peripheral blood mononuclear cells from four healthy donors (#A, #B, #C, and #D) were cultured for 48 hours in standard culture conditions. The cells were immuno-stained and identified based on the marker expression detailed in Materials and Methods on day 2 and analyzed by flow cytometry on day 3. (**A**) The monocytes/macrophages count, along with (**B**) their activation status (HLA-DR expression), were evaluated. In (**A**), each histogram shows the mean ± S.E.M. of cell count obtained for each donor. Each data point (black dot) represents the average of triplicates per donor. In (**B**), each histogram displays the mean ± S.E.M. of the median of fluorescence intensity (MFI) for the HLA-DR expression per donor, with each data point (black dot) representing the average of triplicates per donor. Dotted lines are drawn to highlight the effect of 2LEID-N-9 in comparison with the Veh. Of note, and for indicative and explorative purpose, Wilcoxon signed rank test was performed, and the results were not significant, with the following indicative p values: **A**) p = 0,25, and **B**) p = 0,625.

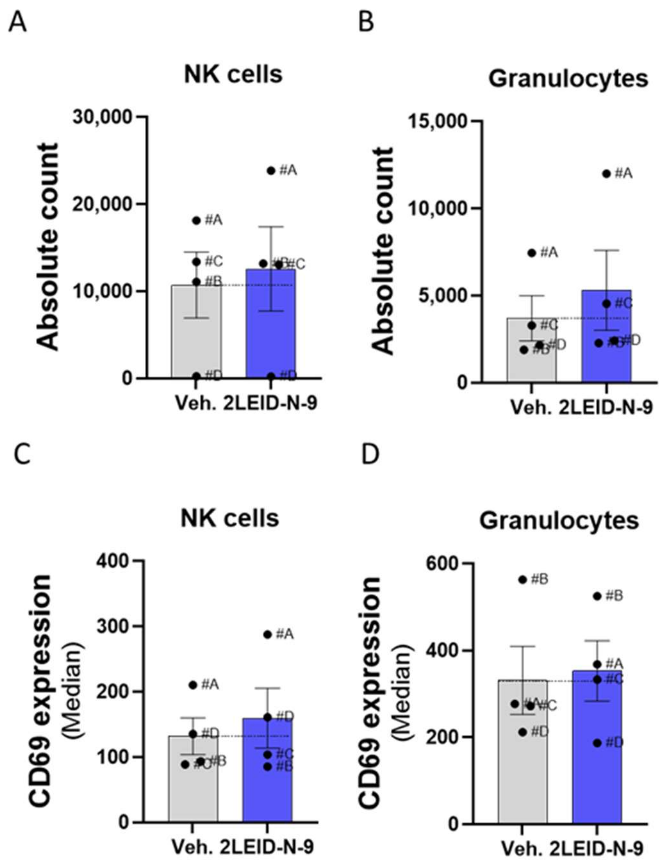

Supplementary Figure S2: The three actives from 2LEID-N-9; IL-12 (5 CH), IFN-γ (6 CH) and TNF-α (5 CH) from 2LEID-N-9 displayed immunomodulatory effects on natural killers (NK) and granulocytes in vitro. Human peripheral blood mononuclear cells (PBMCs) from four healthy donors (#A, #B, #C and #D) were cultured for 48 hours in standard culture conditions. The cells were immuno-stained and identified based on the marker expression detailed in Material and Methods on day 2 and analyzed by flow cytometry on day 3. (**A-B**) The cell count, along with (**C-D**) their activation status (CD69 expression), were evaluated. In (**A-B**), each histogram shows the mean ± standard error of the mean (S.E.M.) of cell count obtained for each donor. Each data point (black dot) represents the average of triplicates per donor. In (**C-D**), each histogram displays the mean ± S.E.M. of the MFI for the CD69 expression per donor, with each data point (black dot) representing the average of triplicates per donor. Dotted lines are drawn to highlight the effect of 2LEID-N-9 in comparison with the Veh. Of note, and for indicative and explorative purpose, Wilcoxon signed rank test was performed, and the results were not significant, with the following indicative *p* values: **A**) *p* = 0,625; **B**) *p* = 0,125; **C**) *p* = 0,25; and **D**) *p* = 0,625

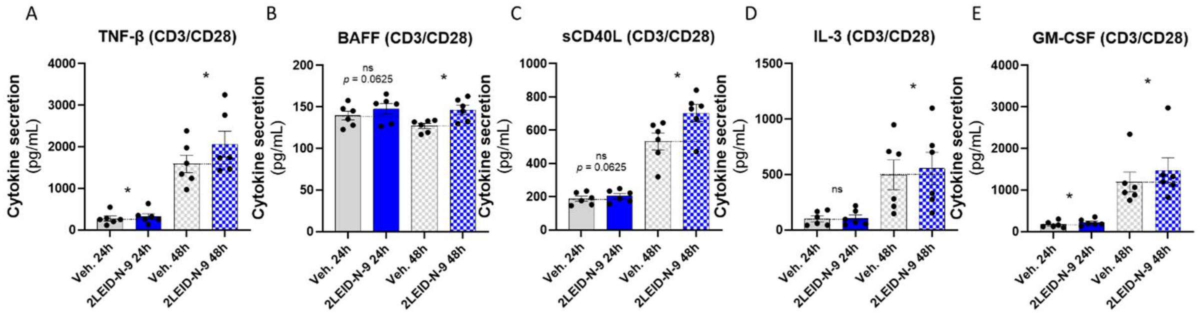

Supplementary Figure S3. The three actives from 2LEID-N-9; IL-12 (5 CH), IFN-γ (6 CH), and TNF-α (5 CH) increased the *in vitro* secretion of some cytokines in Human peripheral blood mononuclear cells (PBMCs) from healthy donors, when activated with CD3/CD28. PBMCs from 6 healthy donors were incubated with the vehicle (Veh.) or with 2LEID-N-9, in the presence of CD3/CD28 antibodies (1 μg/mL and 2 μg/mL, respectively), and the secretion of a panel of five cytokines was assessed in the supernatants (SNs) by enzyme-linked immunosorbent assay (ELISA) after either a 24-hour or a 48-hour incubation period. The secretion of (**A**) TNF-β, (**B**) BAFF, (**C**) sCD40L, (**D**) IL-3, and (**E**) GM-CSF was evaluated. Data are shown as mean ± S.E.M. of the cytokine secretion in pg/mL, obtained for $n = 6$ donors. Each dot represents the mean measure of one duplicate per donor. Dotted lines are drawn to highlight the effect of 2LEID-N-9 in comparison with the Veh. Wilcoxon matched-pairs signed rank tests have been done on the data: * $p < 0.05$; ns: non-significant.

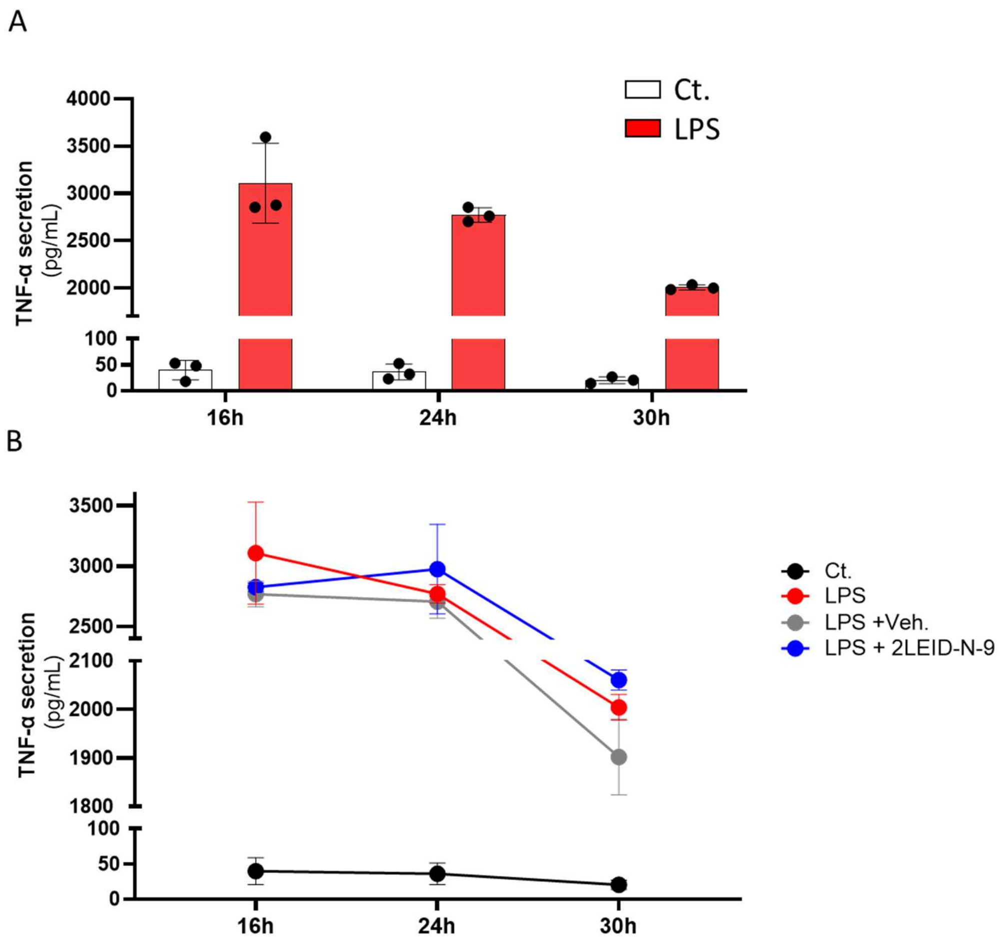

Supplementary Figure S4. Effect of lipopolysaccharide (LPS) on tumor necrosis factor-α (TNF-α) secretion in the human monocytic cell line THP-1. (**A**) THP-1 cells were either left untreated (control; Ct., white histograms), or stimulated with 1 µg/mL LPS (red histograms) at three different time points (16, 24, and 30 hours). The supernatants (SNs) were collected and stored at -80 °C until needed. (**B**) Another representation of the TNF-α secretion, in the same Ct. and LPS conditions, in the presence of either the Veh. or 2LEID-N-9 (see the respective curves in the legend). In these conditions, THP-1 cells were pre-treated with either the Veh. (grey lines) or 2LEID-N-9 (blue lines) for 30 minutes, followed by stimulation with 1 µg/mL LPS. The levels of TNF-α were quantified using enzyme-linked immunosorbent assay (ELISA). Data are shown as mean ± standard deviation (S.D.) of the cytokine secretion (in pg/mL), obtained for $n = 3$ replicates.

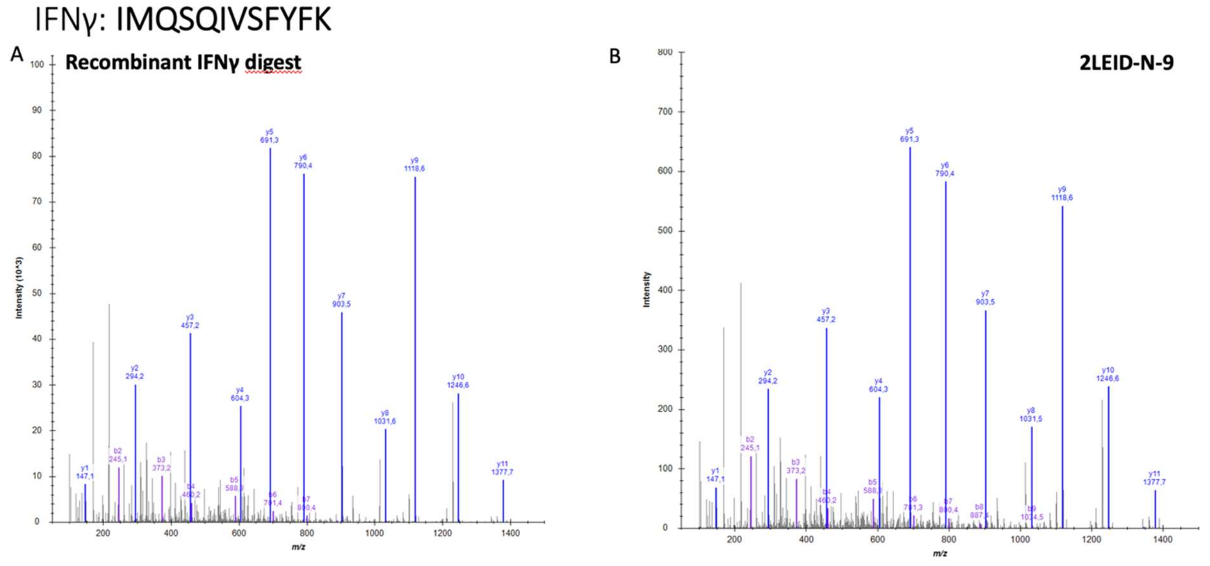

Supplementary Figure S5. MS/MS spectra acquired for IMQSQIVSFYFK proteotypic peptide from interferon-γ (IFN-γ) with y/b ions in (**A**) standard recombinant protein, (**B**) sample 2LEID-N-9. Figure shows the matched fragments confirming specific peptide presence in 2LEID-N-9.

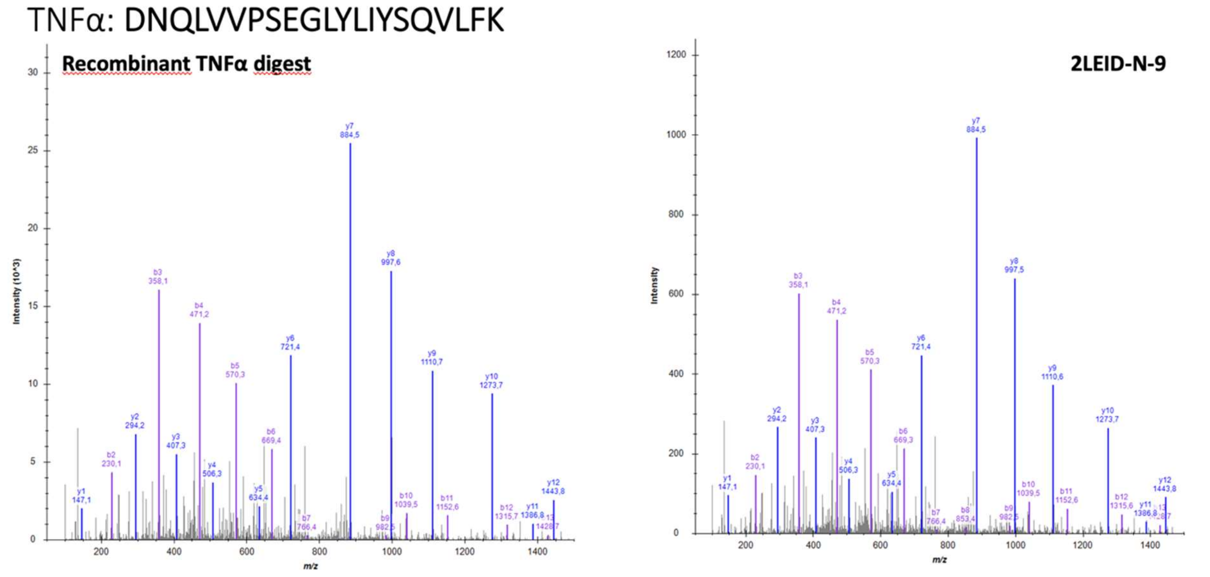

Supplementary Figure S6. MS/MS spectra acquired for DNQLVVPSEGLYLIYSQVLFK proteotypic peptide from tumor necrosis factor-α (TNF-α) with y/b ions in, (**A)** standard recombinant protein, (**B**) sample 2LEID-N-9. Figure shows the matched fragments confirming specific peptide presence in 2LEID-N-9.

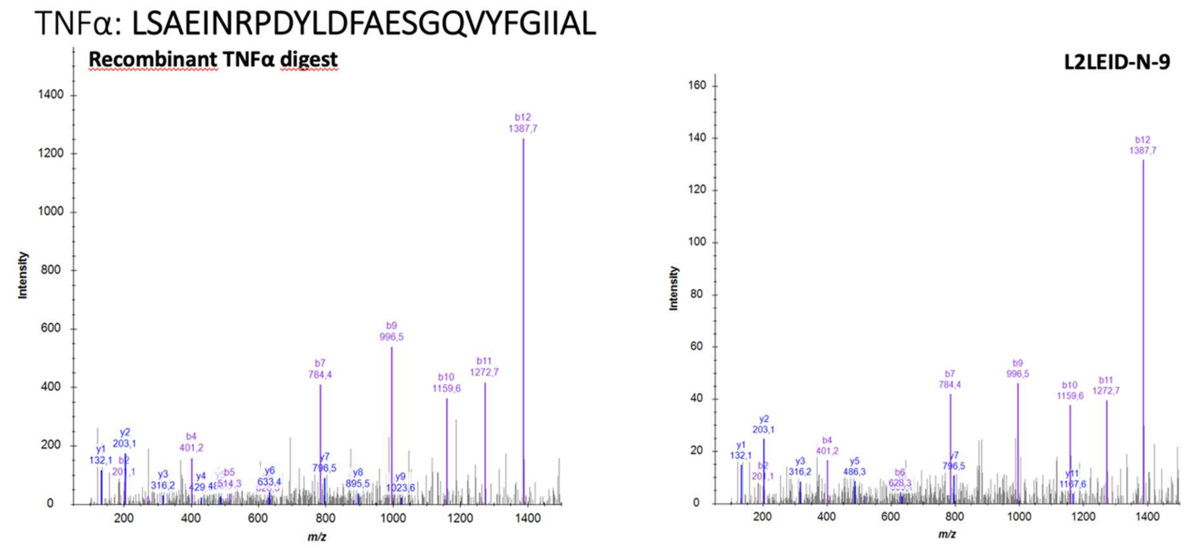

Supplementary Figure S7. MS/MS spectra acquired for LSAEINRPDYLDFAESGQVYFGIIALK proteotypic peptide from tumor necrosis factor-*α* (TNF-*α*) with y/b ions in, (**A)** standard recombinant protein, (**B**) sample 2LEID-N-9. Figure shows the matched fragments confirming specific peptide presence in 2LEID-N-9.

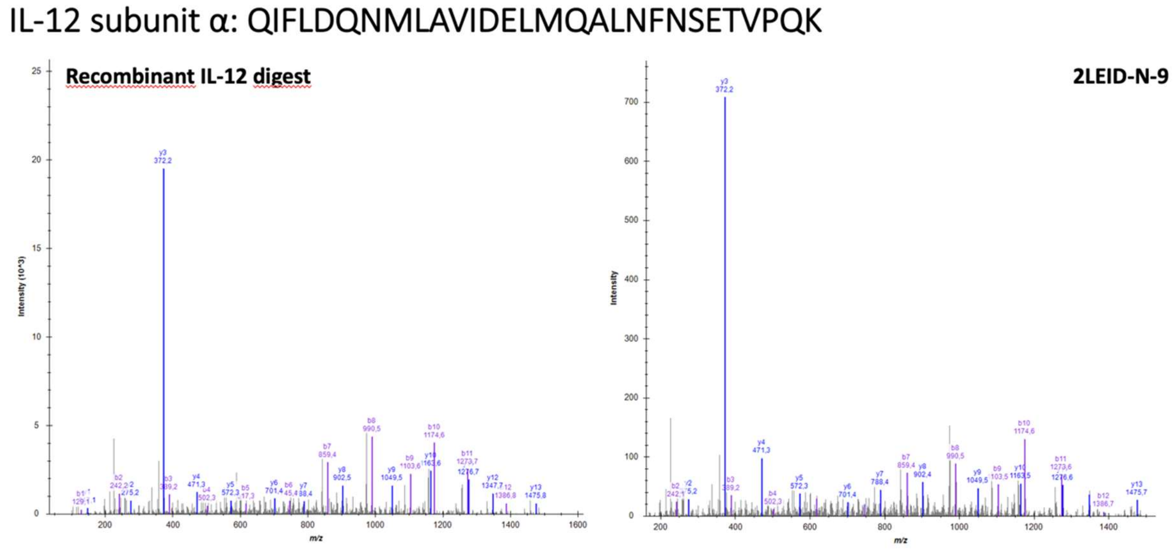

Supplementary Figure S8. MS/MS spectra acquired for QIFLDQNMLAVIDELMQALNFNSETVPQK proteotypic peptide from interleukin-12 subunit alpha (IL-12α) with y/b ions (**A**) Standard recombinant protein. (**B)** Sample 2LEID-N-9. Figure shows the matched fragments confirming specific peptide presence in 2LEID-N-9.

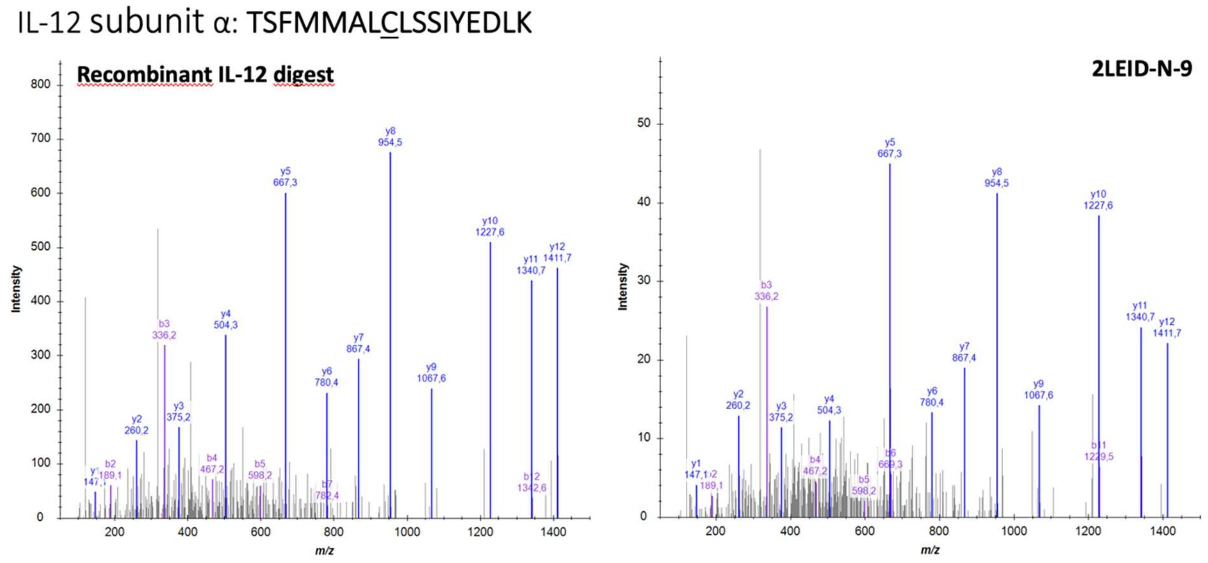

Supplementary Figure S9. MS/MS spectra acquired for TSFMMALCLSSIYEDLK proteotypic peptide from interleukin-12 subunit alpha (IL-12α) with y/b ions (**A**) Standard recombinant protein. (**B)** Sample 2LEID-N-9. Figure shows the matched fragments confirming specific peptide presence in 2LEID-N-9.

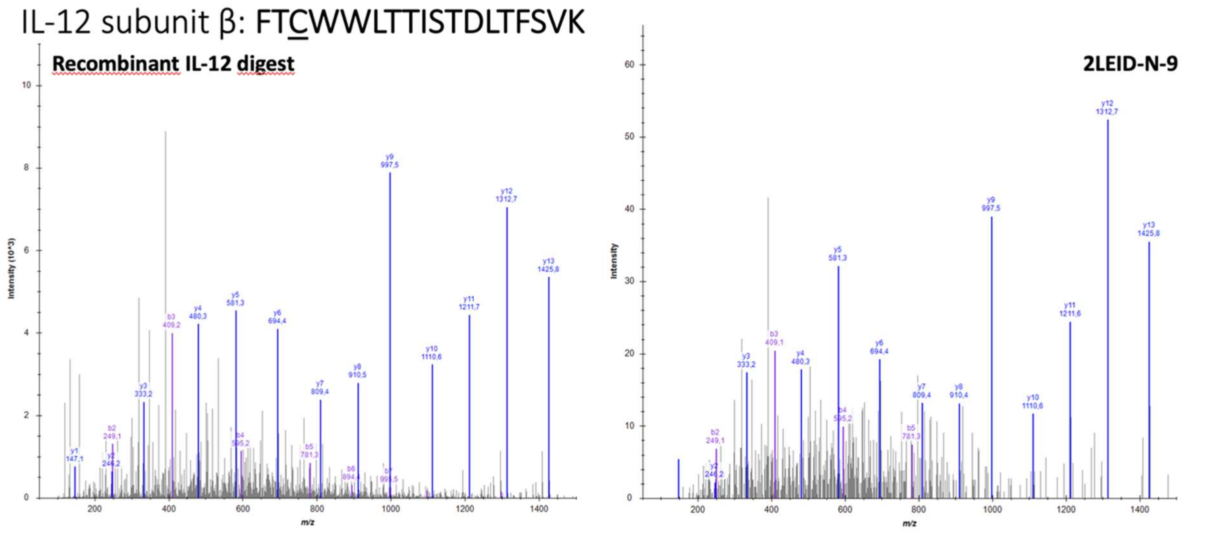

Supplementary Figure S10. MS/MS spectra acquired for FTCWWLTTISTDLTFSVK (carbamydomethil cystein modification in C3) proteotypic peptide from interleukin-12 subunit beta (IL-12β) with y/b ions (**A**) Standard recombinant protein. (**B**) Sample 2LEID-N-9. Figure shows the matched fragments confirming specific peptide presence in 2LEID-N-9.

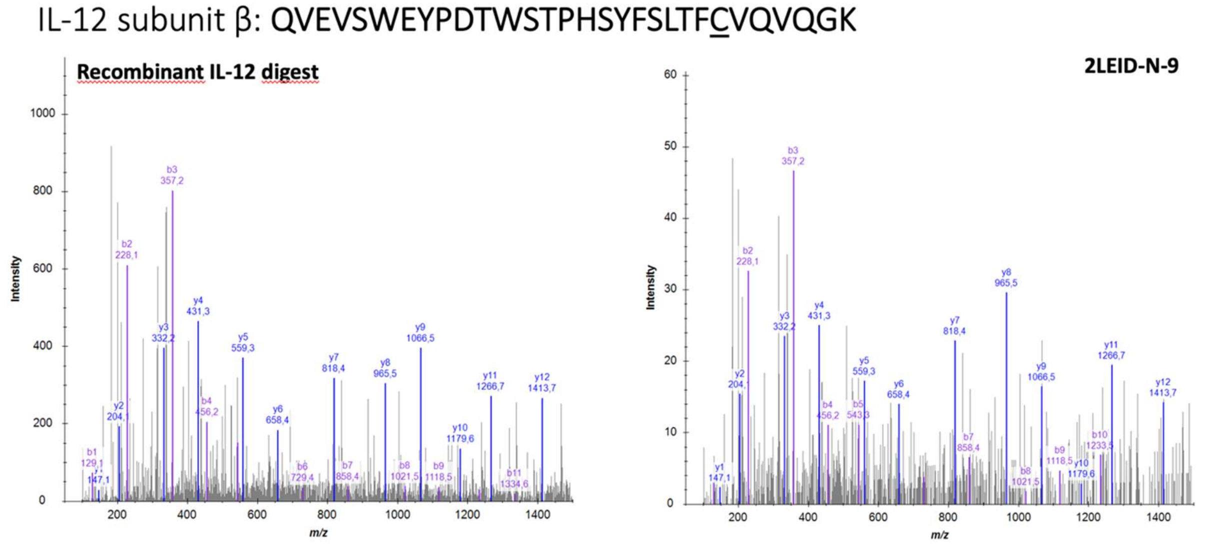

Supplementary Figure S11. MS/MS spectra acquired for FTCWWLTTISTDLTFSVK (carbamydomethil cystein modification in C3) proteotypic peptide from interleukin-12 subunit beta (IL-12β) with y/b ions (**A**) Standard recombinant protein. (**B**) Sample 2LEID-N-9. Figure shows the matched fragments confirming specific peptide presence in 2LEID-N-9.

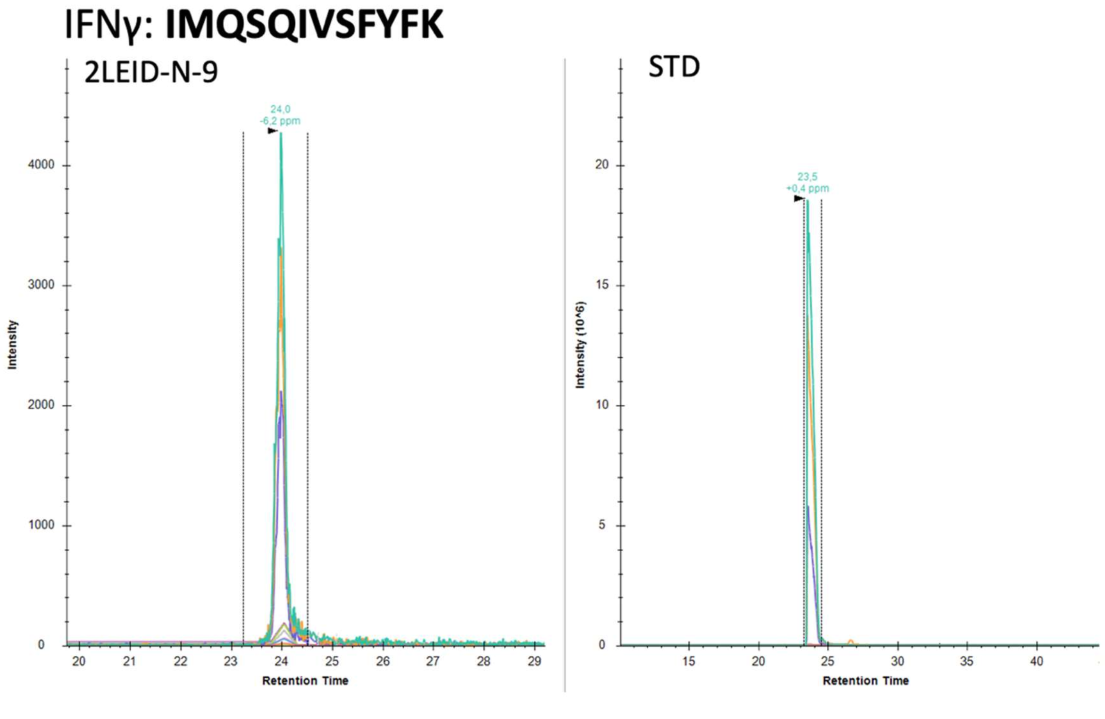

Supplementary Figure S12. Extracted ion chromatogram of the peptides identified from interferon-γ (IFN-γ) in 2LEID-N-9 vs standard (STD) recombinant protein, with false discovery rate (FDR) 1%.

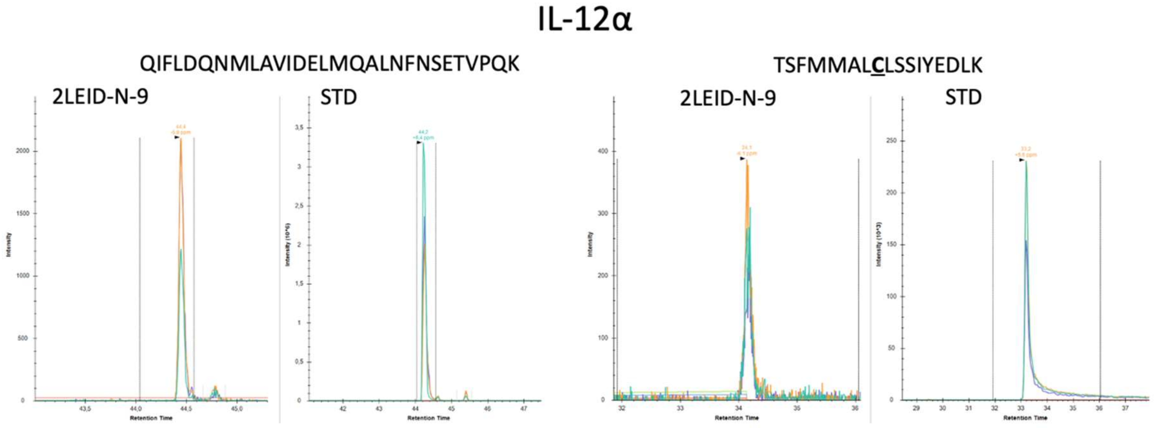

Supplementary Figure S13. Extracted ion chromatogram of the two peptides identified from interleukin-12 subunit alpha (IL-12α) in 2LEID-N-9 vs standard (STD) recombinant protein, with false discovery rate (FDR) 1%.

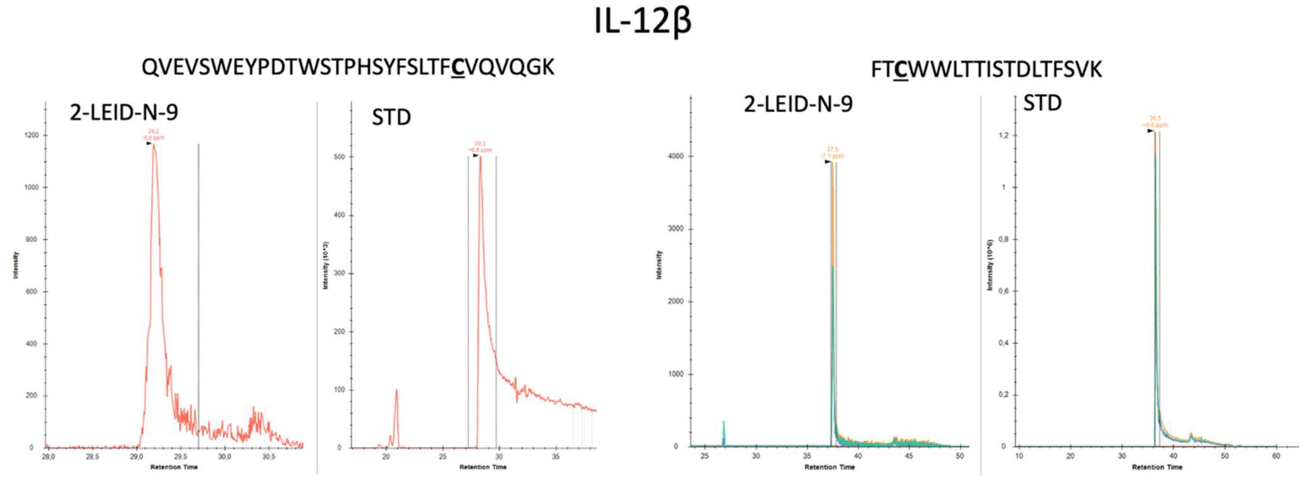

Supplementary Figure S14. Extracted ion chromatogram of the two peptides identified from interleukin-12 subunit beta (IL-12β) in 2LEID-N-9 vs standard (STD) recombinant protein, with false discovery rate (FDR) 1%.

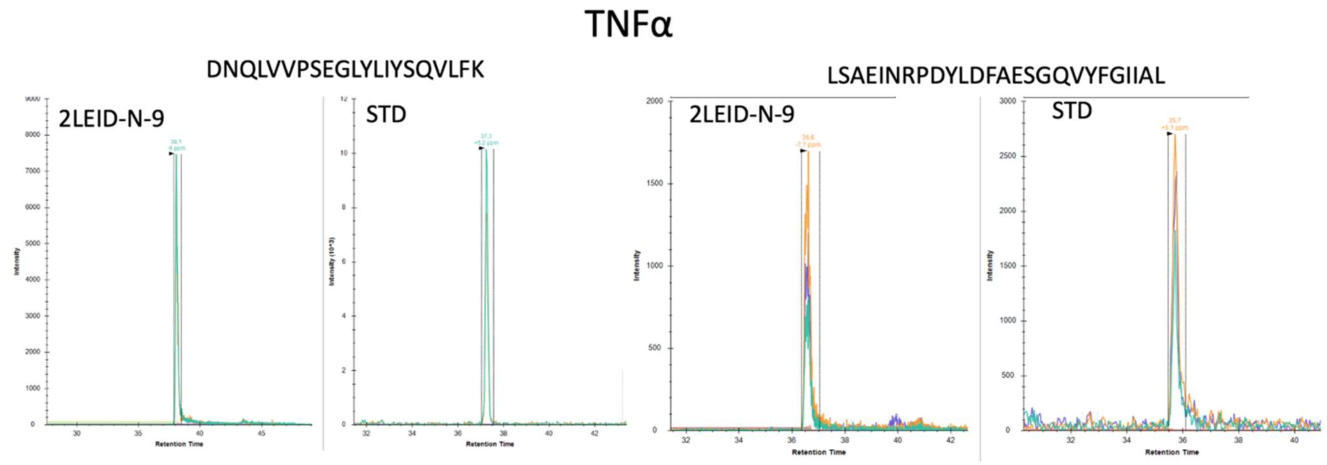

Supplementary Figure S15. Extracted ion chromatogram of the two peptides identified from tumor necrosis factor-α (TNF-α) in 2LEID-N-9 vs standard (STD) recombinant protein, with false discovery rate (FDR) 1%.

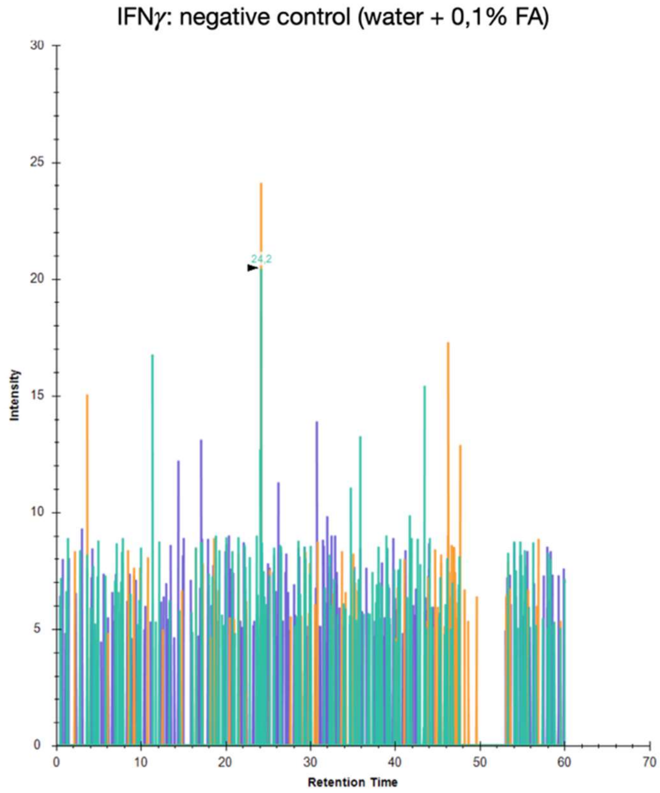

Supplementary Figure S16. LC–HRMS/MS analysis of the negative control (water + 0.1% FA) with peptide signal integration of interferon-γ (IFN-γ), confirming the absence of detectable signal.

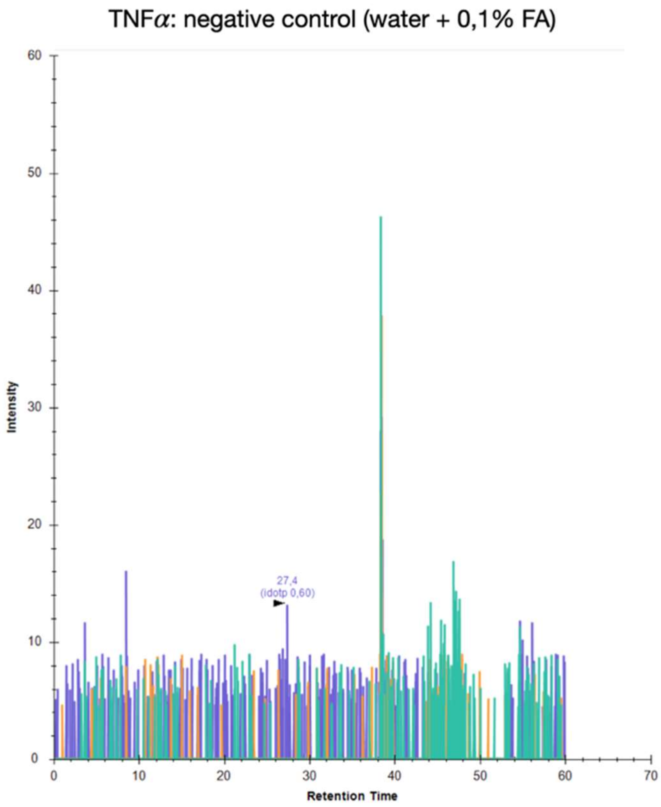

Supplementary Figure S17. LC–HRMS/MS analysis of the negative control (water + 0.1% FA) with peptide signal integration of tumor necrosis factor-α (TNF-α), confirming the absence of detectable signal.

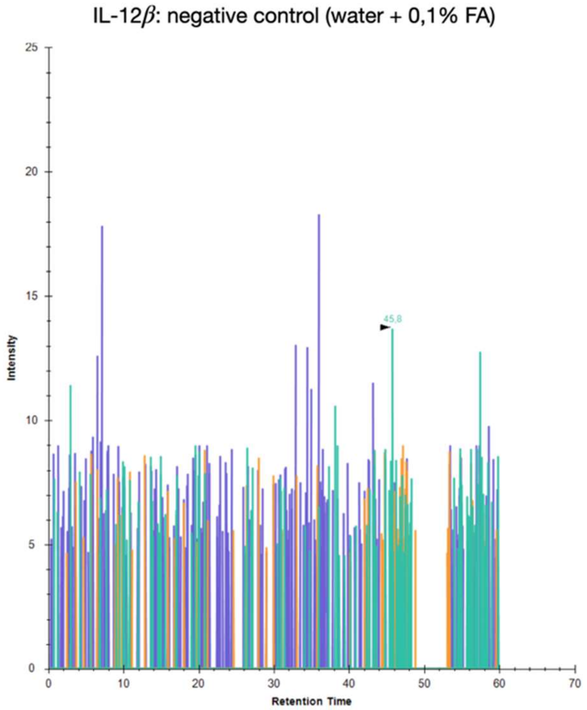

Supplementary Figure S18. LC–HRMS/MS analysis of the negative control (water + 0.1% FA) with peptide signal integration of interleukin-12 subunit beta (IL-12β), confirming the absence of detectable signal.

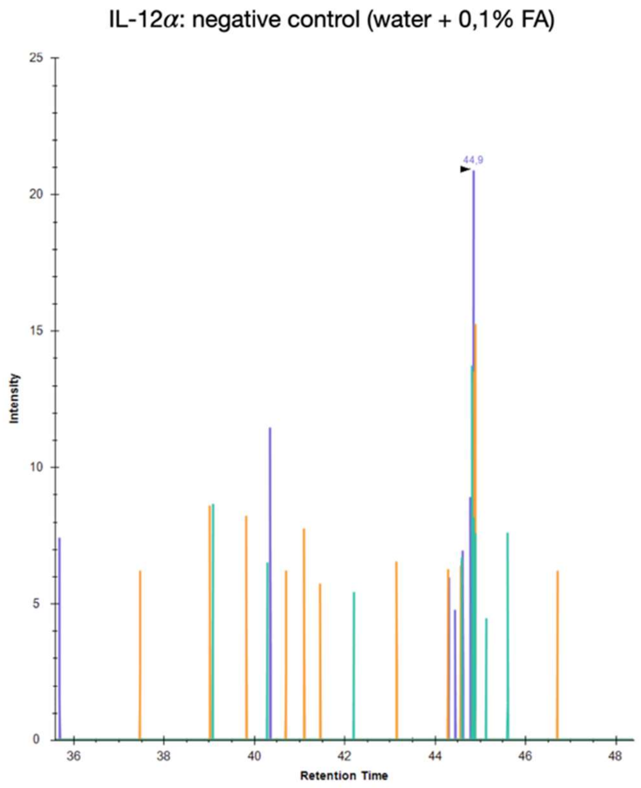

Supplementary Figure S19. LC–HRMS/MS analysis of the negative control (water + 0.1% FA) with peptide signal integration of interleukin-12 subunit alpha (IL-12α) confirming the absence of detectable signal.

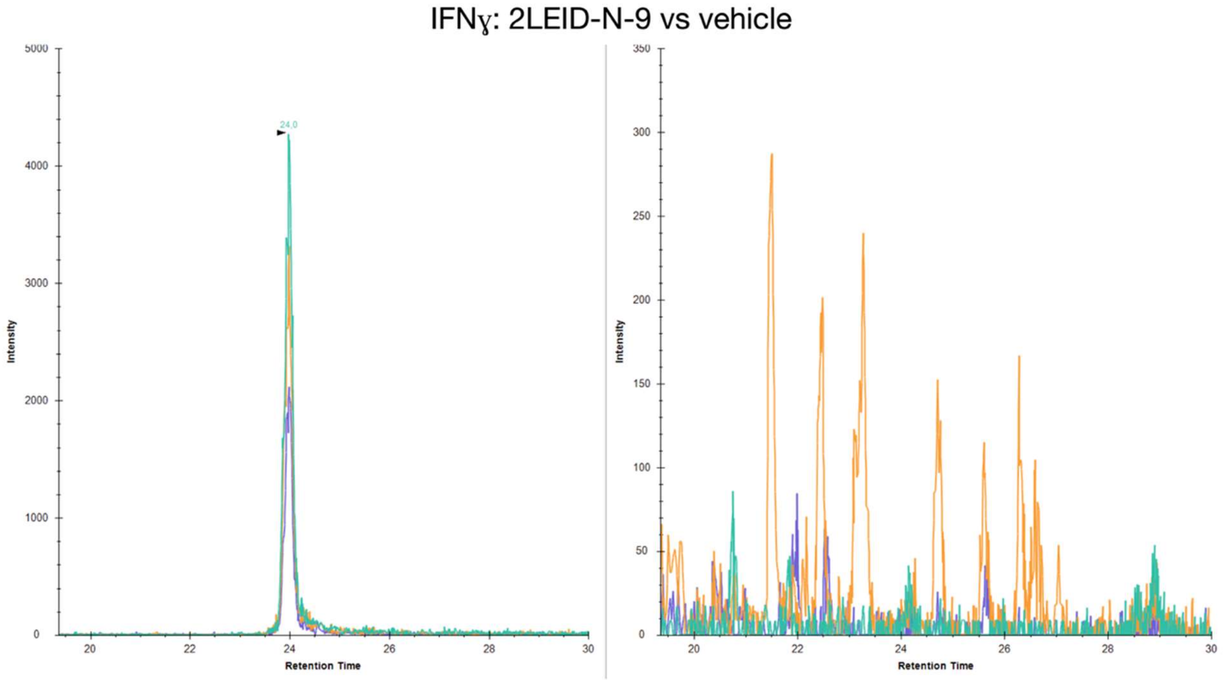

Supplementary Figure S20. LC–HRMS/MS analysis of the vehicle control with peptide signal integration of interferon-γ (IFN-γ) (on the right) vs 2LEID-N-9 sample (on the left), confirming the protein detection in samples.

TNFα: 2LEID-N-9 vs vehicle

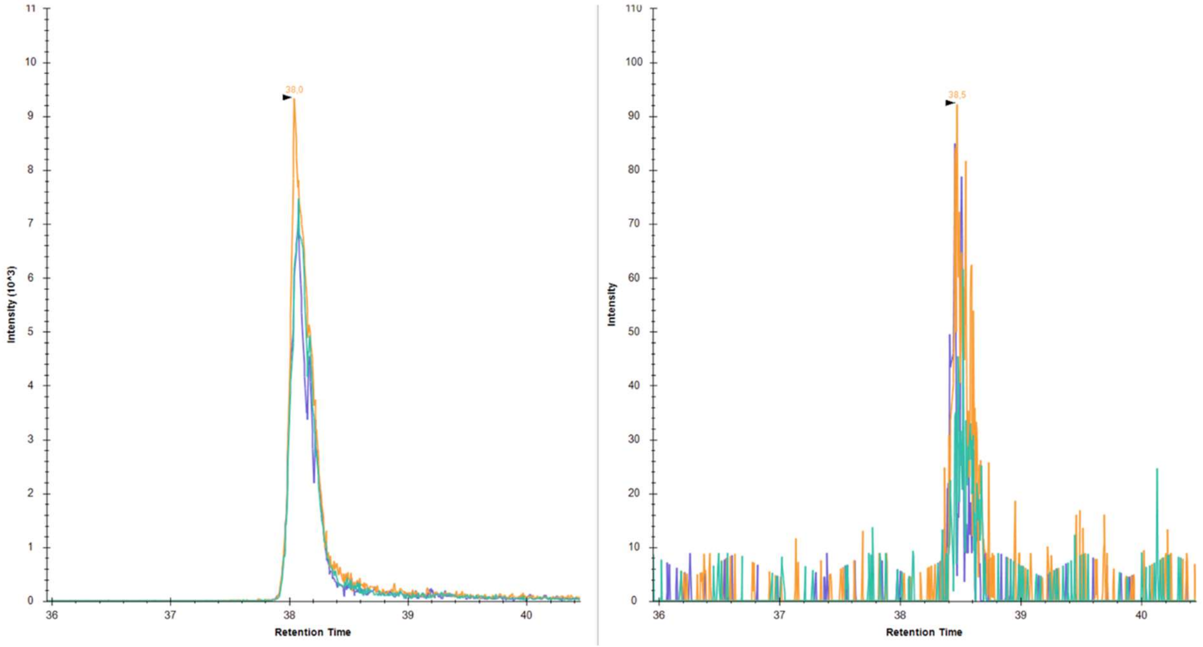

Supplementary Figure S21. LC–HRMS/MS analysis of the vehicle control with peptide signal integration of tumor necrosis factor-α (TNF-α) vs 2LEID-N-9 sample, confirming the protein detection in samples.

IL12α: 2LEID-N-9 vs vehicle

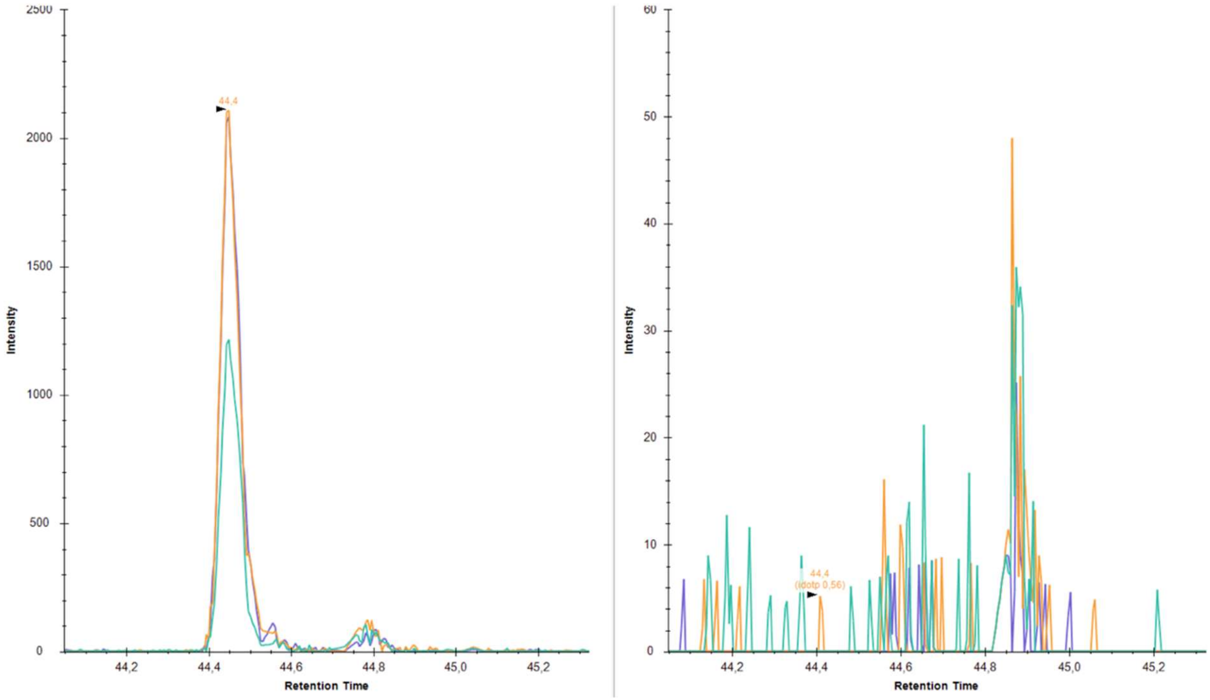

Supplementary Figure S22. LC–HRMS/MS analysis of the vehicle control with peptide signal integration of interleukin 12 (IL12- α) vs 2LEID-N-9 sample, confirming the protein detection in samples.

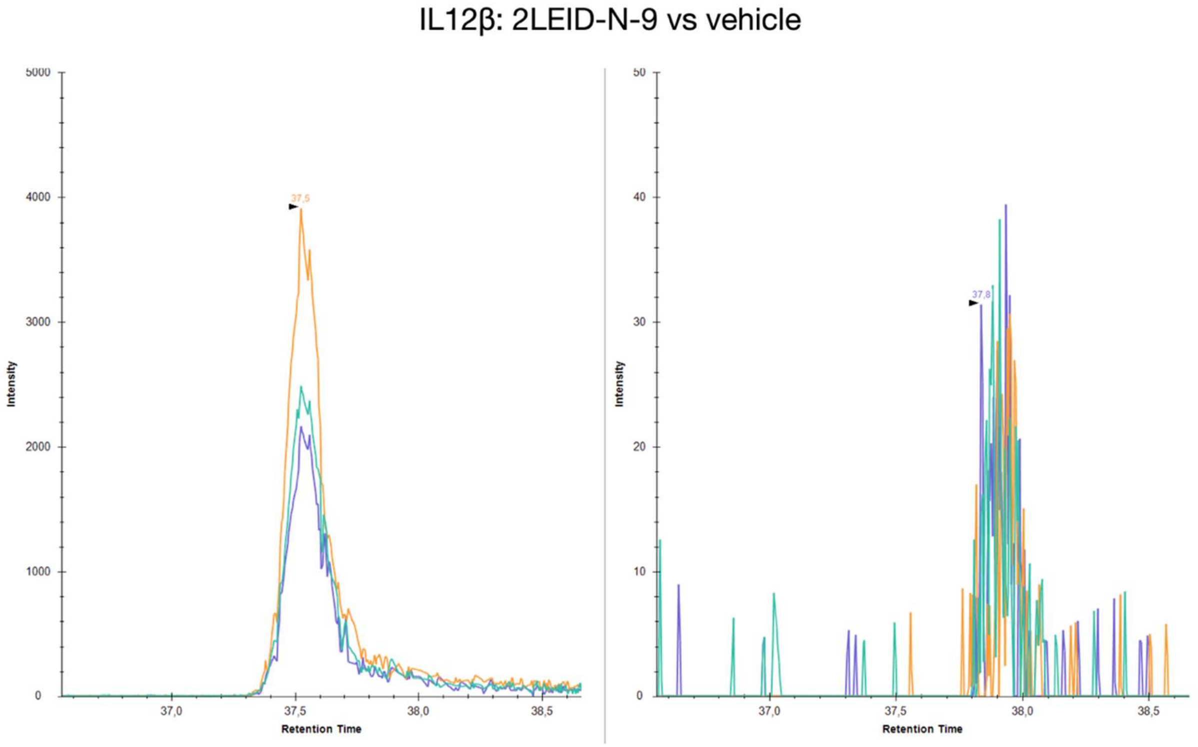

Supplementary Figure S23. LC–HRMS/MS analysis of the vehicle control with peptide signal integration of interleukin-12 subunit beta (IL-12β) vs 2LEID-N-9 sample, confirming the protein detection in samples.
